# Functionalized Biodegradable Polymers via Termination of Ring-Opening Polymerization by Acyl Chlorides

**DOI:** 10.3390/polym13060868

**Published:** 2021-03-11

**Authors:** Ilya Nifant’ev, Andrey Shlyakhtin, Vladimir Bagrov, Evgeny Shaputkin, Alexander Tavtorkin, Pavel Ivchenko

**Affiliations:** 1 Chemistry Department, M.V. Lomonosov Moscow State University, 1–3 Leninskie Gory, 119991 Moscow, Russia; shlyahtinav@mail.ru (A.S.); vlabag@yandex.ru (V.B.); evgeny.shaputkin@yandex.ru (E.S.); phpasha1@yandex.ru (P.I.); 2 Laboratory of Organometallic Catalysis, A.V. Topchiev Institute of Petrochemical Synthesis RAS, 29 Leninsky Pr., 119991 Moscow, Russia; tavtorkin@yandex.ru; 3 Faculty of Chemistry, National Research University Higher School of Economics, 20 Miasnitskaya Str., 101000 Moscow, Russia

**Keywords:** conjugation, coordination catalysis, maleimide, NHS, *N*-hydroxysuccinimide, polycaprolactone, polyesters, polylactide, polyphosphoesters, ring-opening polymerization

## Abstract

Aliphatic polyesters are an important class of polymeric materials for biomedical applications due to their versatile and tunable chemistry, biocompatibility and biodegradability. A capability of direct bonding with biomedically significant molecules, provided by the presence of the reactive end functional groups (FGs), is highly desirable for prospective polymers. Among FGs, N-hydroxysuccinimidyl activated ester group (NHS) and maleimide fragment (MI) provide efficient covalent bonding with –NH– and –SH containing compounds. In our study, we found that NHS- and MI-derived acyl chlorides efficiently terminate living ring-opening polymerization of ε-caprolactone, *L*-lactide, ethyl ethylene phosphonate and ethyl ethylene phosphate, catalyzed by 2,6-di-*tert*-butyl-4-methylphenoxy magnesium complex, with a formation of NHS- and MI-functionalized polymers at a high yields. Reactivity of these polymers towards amine- and thiol-containing model substrates in organic and aqueous media was also studied.

## 1. Introduction

For contemporary pharmacy, tissue engineering and surgery the challenge is to step up implementation of the articles made from biocompatible and biodegradable polymers [1,2,3,4]. Besides the basic functions such as membrane permeability, mechanical strength and histocompatibility, a capability of direct binding with biomedically significant molecules such as drugs, hormones, growth and coagulation factors, enzymes, etc. would be highly desirable for prospective polymer-based articles [5,6,7,8,9,10,11,12,13].

**Scheme 1:**
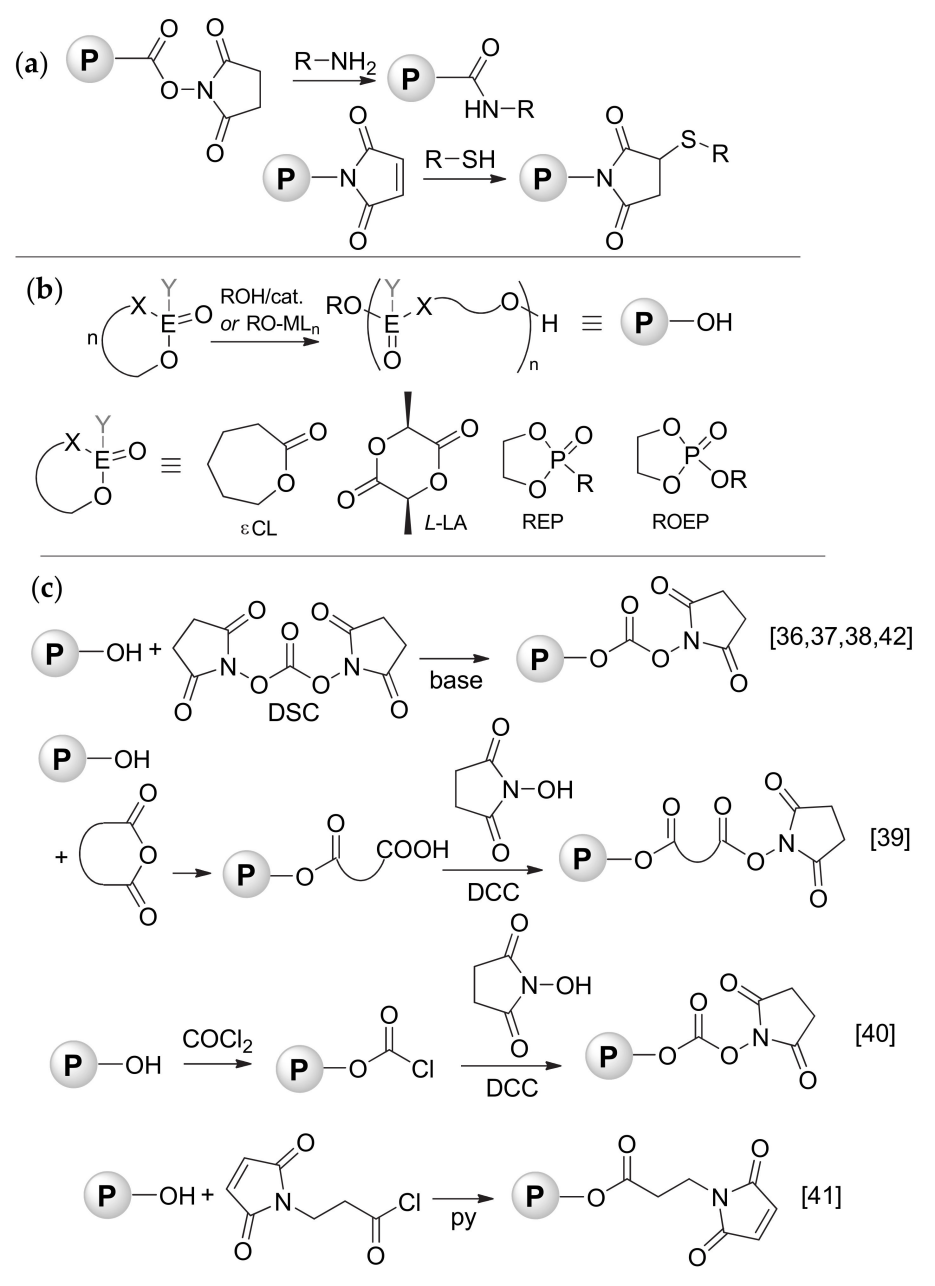
(**a**) Reactivity of NHS (N-hydroxysuccinimidyl) and MI (maleimide) end groups; (**b**) ring-opening polymerization (ROP) and common cyclic substrates; (**c**) known methods of the synthesis of NHS- and MI-functionalized polymers [36,37,38,39,40,41,42,43,44,45].

Most of biomedically significant molecules contain –NH– or –SH fragments by which the chemical binding with polymer can be realized if the event that polymer contains reactive functional groups (FGs) [5,9,14]. Among FGs, N-hydroxysuccinimidyl activated ester group (NHS) and maleimide fragment (MI) were successfully applied for covalent bonding with –NH– and –SH containing compounds, respectively (Scheme 1a) [12,15,16,17,18,19,20,21,22,23,24,25].

Ring-opening polymerization of cyclic esters, phosphates and phosphonates (Scheme 1b) [26,27,28,29,30,31,32,33,34,35] results in the formation of polymers containing –OH end group. To date, a number of methods of the functionalization of –OH end group in separated polymers with a formation of NHS or MI derivatives have been implemented [36,37,38,39,40,41,42,43]. These methods are based on common protocols of the acylation of alcohols (Scheme 1c).

**Scheme 2:**
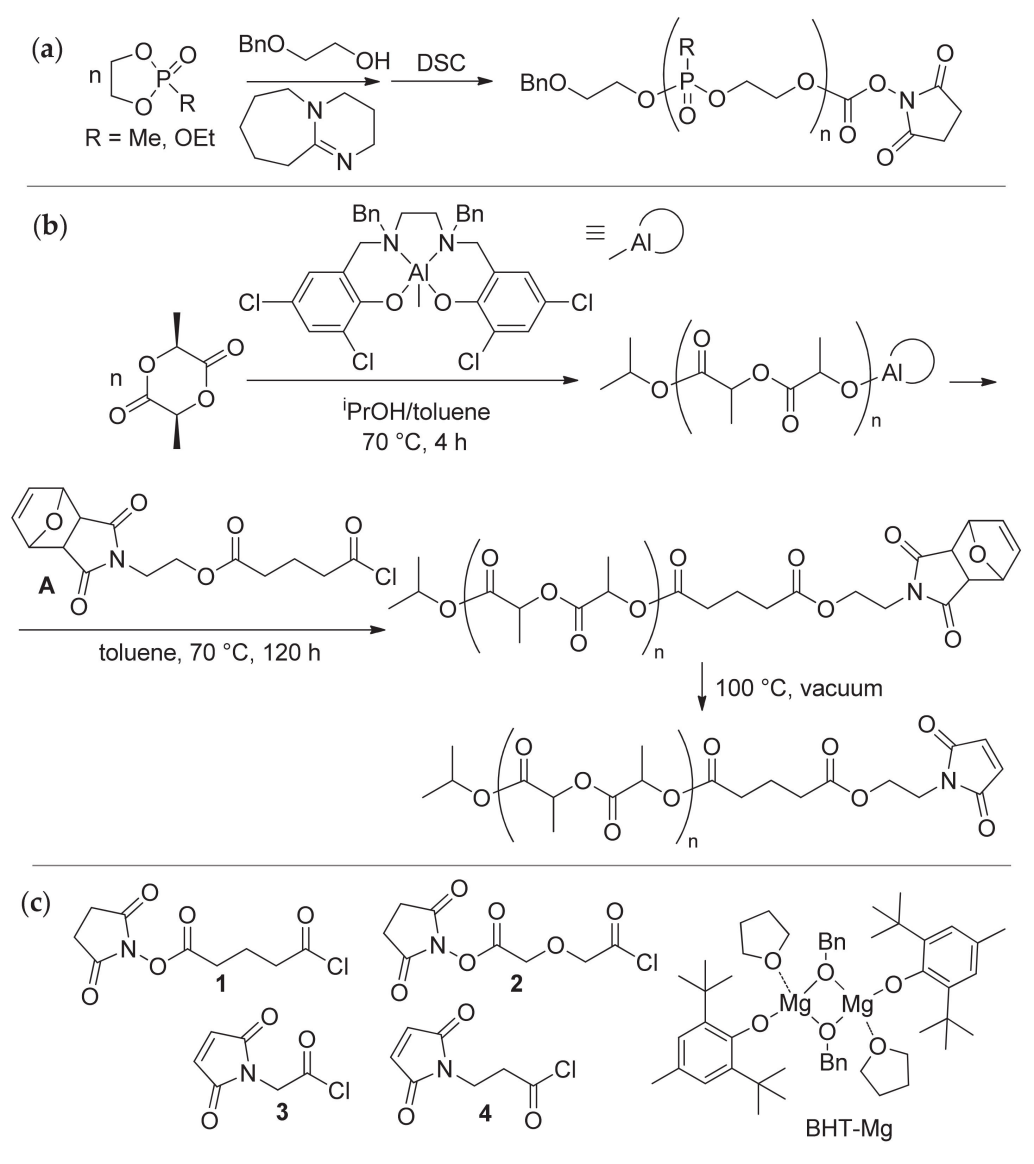
(**a**) NHS functionalization of “living” polyphosphates and polyphosphonates [37,44]; (**b**) MI functionalization of “living” poly(l-LA) [42,46]; (**c**) NHS and MI substituted acyl chlorides and BHT-Mg catalyst used in the synthesis of NHS- and MI-terminated polymers [this work].

The methods presented in Scheme 1 do not necessarily lead to polymers that have 100% degree of functionalization even using large surpluses of the reagents and long reaction times. Considering the “living” nature of the ROP, catalyzed by a number of both organocatalysts and coordination catalysts, polymer functionalization involving activated chain-end alkoxy group seems reasonable. This idea was realized by Wurm et al. by the example of termination of 1,8-diazabicyclo[5.4.0]undec-7-ene (DBU)/BnO(CH_2_)_2_OH catalyzed ROP of ethylene phosphonate or ethylene phosphate using *N,N*′-disuccinimidyl carbonate (DSC) [37,44] (Scheme 2a). Stanford and Dove implemented this approach in ROP of l-lactide (l-LA), catalyzed by Al salen complexes and terminated by acyl chloride **A** with subsequent retro-Diels-Alder reaction (Scheme 2b), however, such functionalization required long time at elevated temperatures (120 h, 70 °C) [45,46].

Recently, we demonstrated high efficiency of alkoxy Mg complexes of 2,6-di-*tert*-butyl-4-methylphenol (BHT-H) in “living” ROP of different cyclic esters, phosphates and phosphonates [47,48,49,50,51,52,53,54,55]. In this paper, we report that “living” alkoxy Mg-BHT chain end can be efficiently terminated under mild reaction conditions by acyl chlorides **1**–**4** (Scheme 2c) with simultaneous insertion of NHS and MI fragments, suitable for eventual interactions with –NH– and –SH containing compounds. NHS- and MI-functionalized polymers were studied in model reactions with isobutylamine (^i^BuNH_2_) and methyl 2-mercaptoacetate (HSCH_2_COOMe) in organic and aqueous media to evaluate the potential of the chemical binding of these polymers with biomedically significant molecules.

## 2. Materials and Methods

### 2.1. General Experimental Remarks

All of the synthetic and polymerization experiments were performed under a purified argon atmosphere. Tetrahydrofuran (THF), 1,4-dioxane, diethyl ether (Et_2_O) and *N,N,N*-triethylamine (Et_3_N) (Merck, Darmstadt, Germany) were refluxed with Na/benzophenone and distilled prior to use. CH_2_Cl_2_ (Prime Chemicals Group, Moscow, Russia) was washed with aqueous Na_2_CO_3_, stirred with CaCl_2_ powder, refluxed over CaH_2_ for 8 h and distilled. Acetonitrile (MeCN, Merck, Darmstadt, Germany) was stored over K_2_CO_3_, refluxed, and distilled from CaH_2_. ε-Caprolactone (εCL, Merck, Darmstadt, Germany) was distilled prior to use under argon over CaH_2_. l-Lactide (l-LA, Merck, Darmstadt, Germany) was purified by recrystallization from toluene and sublimation. *N,N*-dimethylformamide (DMF), ethyl acetate (EtOAc), dimethoxymethane (DMM), acetyl chloride (AcCl), *N*-hydroxysuccinimide, succinic anhydride (SA), *N,N*-dimethylaminopyridine (DMAP), HSCH_2_COOMe, ^i^BuNH_2_, *N,N′*-dicyclohexylcarbodiimide (DCC), DSC, oxalyl chloride (C_2_O_2_Cl_2_), thionyl chloride (SOCl_2_), glutaric anhydride, and glycine were used as purchased (Merck, Darmstadt, Germany). 1,4-dioxane-2,6-dione [56], 2-(2,5-dioxo-2,5-dihydro-1*H*-pyrrol-1-yl)acetic acid (glycine maleimide) [57], 3-(2,5-dioxo-2,5-dihydro-1*H*-pyrrol-1-yl)propanoic acid [58], 2-ethyl-1,3,2-dioxaphospholane 2-oxide (ethyl ethylene phosphonate, EtEP) [59], 2-ethoxy-1,3,2-dioxaphospholane 2-oxide (ethyl ethylene phosphate, EtOEP) [60], 1,5,7-triazabicyclo[4.4.0]dec-5-ene (TBD) [61], and [(BHT)Mg(μ-OBn)(THF)]_2_ (BHT-Mg) [50] were synthesized according to the literature procedures. Benzoylated dialysis tubing D2272 (Merck, Darmstadt, Germany) was used for synthesis and purification of functionalized polymers.

CDCl_3_ (D 99.8%, Cambridge Isotope Laboratories, Inc., Cambridge, MS, USA) was distilled over P_2_O_5_ and stored over 4 Å molecular sieves. DMSO-d_6_ (D 99.8%, Cambridge Isotope Laboratories, Inc., Cambridge, MS, USA) was used as purchased. The ^1^H (400 MHz), ^13^C (101 MHz) and ^31^P (162 MHz) NMR spectra were recorded on a Bruker AVANCE 400 spectrometer (Bruker, Billerica, MS, USA) at 20 °C. The chemical shifts were reported relative to the solvent residual peaks (*δ* = 7.26 ppm for CDCl_3_ and *δ* = 2.50 ppm for DMSO-d_6_).

Size exclusion chromatography (SEC) was performed on an Agilent PL-GPC 220 chromatograph (Agilent Technologies, Santa Clara, CA, USA) equipped with a PLgel column, using THF (for εCL, l-LA polymers) or DMF (for EtEP and EtOEP polymers) as the eluents (1 mL/min). The measurements were recorded at 40 °C with universal calibration using polystyrene standards (εCL and l-LA polymers, corrected by the factors of 0.56 for poly(*ε*CL) and 0.58 for poly(l-LA)) or poly(ethylene glycol) standards (for EtEP and EtOEP polymers).

Elemental analysis (C, H, N, O) was performed on a Perkin Elmer Series II CHNS/O Analyzer 2400 (Perkin Elmer, Waltham, MS, USA).

### 2.2. Synthesis of NHS- and MI-Functionalized Acyl Chlorides

Except new compound **2**, acyl chlorides **1**, **3** and **4** have been obtained previously and typically used in situ, without separation and purification [41,62,63,64]. Out of these compounds, only acyl chloride **4** had been characterized by ^1^H and ^13^C NMR spectroscopy. In this section, we report NMR spectral data for **1**–**4**, ^1^H and ^13^C NMR spectra of **1**–**4** are presented in Figures S1–S8 in the Supplementary Materials.

#### 2.2.1. Synthesis of 2,5-dioxopyrrolidin-1-yl 5-chloro-5-oxopentanoate **1**

Acyl chloride **1** was synthesized according to the modified literature procedure [61]. *N*-hydroxysuccinimide (3.45 g, 30 mmol) was dissolved in THF (200 mL), DMAP (4.9 g, 40 mmol) was added with stirring. Resulting clear solution was cooled to 5 °C, glutaric anhydride (3.42 g, 30 mmol) in THF solvent (20 mL) was added dropwise. The mixture was allowed to warm to room temperature (RT), stirred for 30 min, and evaporated. EtOAc (100 mL), H_2_O (50 mL) and conc. HCl (7 mL) were added. Organic layer was separated, washed by water, dried over MgSO_4_ and evaporated under reduced pressure (Rotavapor R-100, BÜCHI Labortechnik AG, Flawil, Switzerland). The residue was recrystallized from CHCl_3_ and dried in vacuum (RZ 6 rotary vine pump, Vacuubrand GMBH, Wertheim, Germany). For 5-((2,5-dioxopyrrolidin-1-yl)oxy)-5-oxopentanoic acid the yield was 5.0 g (73%). The acid was dissolved in CH_2_Cl_2_ (30 mL), the solution was cooled to 0 °C, then DMF (5 µL) and C_2_O_2_Cl_2_ (3.9 mL, 45 mmol, 1.5 eq.) were added. After 2 h of stirring, the volatiles were removed under reduced pressure, and the residue was dried to constant weight at 0.01 Torr and ambient temperature. The yield was 4.6 g (62%), yellow solid. For C_9_H_10_ClNO_5_ calculated (%): C, 43.65; H, 4.07; N, 5.66; O, 32.30. Found (%): C, 43.68; H, 4.11; N, 5.69; O, 32.36. ^1^H NMR (CDCl_3_, 20 °C) δ: 3.07 (t, ^3^*J* = 7.1 Hz, 2H); 2.82 (bs, 4H); 2.70 (t, ^3^*J* = 7.1 Hz, 2H); 2.10 (p, ^3^*J* = 7.1 Hz, 2H). ^13^C NMR (CDCl_3_, 20 °C) δ: 173.31; 169.17; 167.72; 45.36; 29.36; 25.64; 20.03.

#### 2.2.2. Synthesis of 2,5-dioxopyrrolidin-1-yl 2-(2-chloro-2-oxoethoxy)acetate **2**

*N*-hydroxysuccinimide (10 mmol, 1.15 g) was dissolved in THF (100 mL), DMAP (1.22 g, 10 mmol) was added with stirring. Resulting clear solution was cooled to 5 °C, 1,4-dioxane-2,6-dione (1.16 g, 10 mmol) in THF solvent (10 mL) was added dropwise. The mixture was allowed to warm to RT and stored for 2 days. The mixture was cooled to 5 °C, DMF (10 µL) and C_2_O_2_Cl_2_ (1.29 mL, 15 mmol, 1.5 eq.) were added within 2 h. The reaction mixture was filtered off, the solution was evaporated under reduced pressure, the solid residue was washed by Et_2_O, dissolved in minimal volume of benzene at 80 °C, filtered, and evaporated under reduced pressure. The residue was washed by Et_2_O and dried to constant weight at 0.01 Torr and ambient temperature. The yield was 0.31 g (25%). Melting point (M. p.) 100–102 °C. For C_8_H_8_ClNO_6_ calculated (%): C, 38.50; H, 3.23; N, 5.61; O, 38.46. Found (%): C, 38.46; H, 3.26; N, 5.62; O, 38.51. ^1^H NMR (CDCl_3_, 20 °C) δ: 4.610 (s, 2H); 4.607 (s, 2H); 2.87 (bs, 4H). ^13^C NMR (CDCl_3_, 20 °C) δ: 171.15; 168.71; 164.96; 75.44; 65.95; 25.69.

#### 2.2.3. Synthesis of 2-(2,5-dioxo-2,5-dihydro-1H-pyrrol-1-yl)acetyl chloride **3**

Acyl chloride **3** was synthesized according to the modified literature procedure [63]. Glycine maleimide (1.55 g, 10 mmol) was refluxed with SOCl_2_ (7.3 mL, 100 mmol, 10 eq.) within 30 min. The volatiles were removed under reduced pressure, toluene (5 mL) was added and removed under reduced pressure. The residue was dried to constant weight at 0.01 Torr and ambient temperature. The yield was 1.7 g (98%), pale yellow oil. For C_6_H_4_ClNO_3_ calculated (%): C, 41.52; H, 2.32; N, 8.07; O, 27.66. Found (%): C, 41.58; H, 2.36; N, 8.02; O, 27.69. ^1^H NMR (CDCl_3_, 20 °C) δ: 6.82 (s, 2H); 4.64 (s, 2H). ^13^C NMR (CDCl_3_, 20 °C) δ: 169.27; 168.90; 134.72; 47.33.

#### 2.2.4. Synthesis of 3-(2,5-dioxo-2,5-dihydro-1H-pyrrol-1-yl)propanoyl chloride **4**

Acyl chloride **3** was synthesized according to the modified literature procedure [64]. 3-(2,5-dioxo-2,5-dihydro-1*H*-pyrrol-1-yl)propanoic acid [58] (0.44 g, 2.6 mmol) was dissolved in CH_2_Cl_2_ (15 mL). DMF (5 µL) was added, the solution was cooled to −10 °C, and C_2_O_2_Cl_2_ (0.33 mL, 3.9 mmol, 1.5 eq.) was added. The reaction mixture was allowed to warm to RT, stirred for 4 h, and evaporated under reduced pressure. The residue was dried to constant weight at 0.01 Torr and ambient temperature. The yield was 0.45 g (92%), yellow-green solid. For C_7_H_6_ClNO_3_ calculated (%): C, 44.82; H, 3.22; N, 7.47; O, 25.59. Found (%): C, 44.88; H, 3.27; N, 7.43; O, 25.65. ^1^H NMR (CDCl_3_, 20 °C) δ: 6.73 (s, 2H); 3.85 (t, ^3^*J* = 6.9 Hz, 2H); 3.24 (t, ^3^*J* = 6.9 Hz, 2H). ^13^C NMR (CDCl_3_, 20 °C) δ: 171.53; 170.09; 134.45; 44.95; 33.17.

### 2.3. Synthesis of NHS- and MI-Functionalized Polymers

#### 2.3.1. Polymerization of εCL with Termination by DSC

A preheated 30 mL glass ampoule was equipped with a magnetic stir bar and a septum and then filled with dry argon. εCL (0.96 mL, 8.6 mmol, 50 eq.) was placed into the ampule, CH_2_Cl_2_ (2.3 mL) was added. The mixture was cooled to 5 °C, and BHT-Mg (73 mg, 0.17 mmol Mg, 1 eq.) in THF solvent (1.0 mL) was added (resulting concentration of εCL was 2 M). After 4 h of stirring, the reaction mixture was cooled to −20 °C, and DSC (218 mg, 0.85 mmol, 5 eq.) in MeCN solvent (3 mL) was added. After 3 h of stirring, the reaction mixture was filtered, the filtrate was evaporated to dryness under reduced pressure, dissolved in CH_2_Cl_2_ (5 mL) and precipitated in dry MeOH (40 mL). The polymer was filtered off and dried to constant weight at 0.01 Torr and ambient temperature. The yield was 0.80 g (81%). ^1^H NMR (CDCl_3_, 20 °C) δ: 7.34 (m, 5H, C_6_*H*_5_); 5.10 (s, 2H, PhC*H*_2_); 4.05 (t, ^3^*J* = 6.7 Hz, 98H, –OC*H*_2_– of poly(εCL)); 2.83 (s, 1.33H, –CH_2_– of NHS); 2.30 (t, ^3^*J* = 6.8 Hz, 99H, C(O)CH_2_ of poly(εCL)); 1.64 (m, 203H, –CH_2_– of poly(εCL)); 1.37 (p, ^3^*J* = 6.8 Hz, 100H, –CH_2_– of poly(εCL)), see Figure S9 in the Supplementary Materials.

#### 2.3.2. Polymerization of εCL with Termination by SA Followed by the Reaction with *N*-hydroxysuccinimide/DCC

A preheated 30 mL glass ampoule was equipped with a magnetic stir bar (Merck, Darmstadt, Germany) and a septum and then filled with dry argon. εCL (0.96 mL, 8.6 mmol, 50 eq.) was placed into the ampule, CH_2_Cl_2_ (2.3 mL) was added. The mixture was cooled to 5 °C, BHT-Mg (73 mg, 0.17 mmol Mg, 1 eq.) in THF solvent (1.0 mL) was added (resulting concentration of εCL was 2 M). After 4 h of stirring, a solution of 68 mg SA (0.68 mmol, 4 eq.) in THF solvent (1.0 mL) was added at 5 °C. After 30 min of stirring at 5 °C, polymer solution was precipitated twice in diethyl ether (30 mL) and subsequently dried to constant weight at 0.01 Torr and RT. The product was dissolved in THF (4 mL), then NHS (98 mg, 0.85 mmol, 5 eq.) in THF solvent (2 mL) and DCC (175 mg, 0.85 mmol, 5 eq.) in THF solvent (2 mL) were added subsequentially. After stirring overnight, the reaction mixture was filtered, the filtrate was precipitated in Et_2_O (50 mL). The polymer was filtered off and dried to constant weight at 0.01 Torr and RT. The yield was 0.75 g (76%). ^1^H NMR (DMSO-d_6_, 20 °C) δ: 7.35 (m, 5H, C_6_*H*_5_); 5.07 (s, 2H, PhC*H*_2_); 3.98 (t, 119H, –OC*H*_2_– of poly(εCL)); 2.92 (t, ^3^*J* = 6.3 Hz, 1.10H, –C(O)(C*H*_2_)_2_C(O)–); 2.80 (s, 2.06H, –CH_2_– of NHS); 2.66 (t, ^3^*J* = 6.3 Hz, 1.10H, –C(O)(C*H*_2_)_2_C(O)–); 2.26 (t, 117H, C(O)CH_2_ of poly(εCL)); 1.54 (m, 239H, –CH_2_– of poly(εCL)); 1.29 (p, 121H, –CH_2_– of poly(εCL)), see Figure S10 in the Supplementary Materials.

#### 2.3.3. Polymerization of εCL with Termination by **1**–**4**

A preheated 30 mL glass ampoule was equipped with a magnetic stir bar and a septum and then filled with dry argon. εCL (2.30 mL, 20.8 mmol, 120 eq.) was placed into the ampule, CH_2_Cl_2_ was added to total volume of 8.4 mL. The mixture was cooled to 5 °C, BHT-Mg (73 mg, 0.17 mmol Mg, 1 eq.) in THF solvent (2.0 mL) was added (resulting concentration of εCL was 2 M). After 4 h of stirring, chloroanhydride (0.69 mmol, 4 eq.) in CH_2_Cl_2_ solvent (2 mL) was added. The mixture was allowed to warm to RT and stirred for 8 h. Reaction mixture was evaporated twice with CH_2_Cl_2_ for removal of THF. The residue was dissolved in CH_2_Cl_2_ (40 mL), washed by 0.7 M aq. HCl, by water, dried over MgSO_4_ and filtered. The filtrate was evaporated to residual volume of 20 mL and poured into Et_2_O (100 mL). The polymer was filtered off and dried to constant weight at 0.01 Torr and RT. The yields were 2.31 (95%), 1.83 (75%), 2.11 (87%), and 1.89 g (78%) for poly(εCL) functionalized by **1**, **2**, **3**, and **4**, respectively. ^1^H NMR (CDCl_3_, 20 °C) δ: 7.31 (m, 5H, C_6_*H*_5_); 5.07 (s, 2H, PhC*H*_2_); 4.02 (t, 299H, –OC*H*_2_– of poly(εCL)); 2.81 (s, 4H, –CH_2_– of NHS); 2.67 (t, ^3^*J* = 7.5 Hz, 2H; –CH_2_CH_2_C*H*_2_C(O)NHS); 2.42 (t, ^3^*J* = 7.5 Hz, 2H; –C*H*_2_CH_2_CH_2_C(O)NHS); 2.27 (t, 296H, C(O)CH_2_ of poly(εCL)); 2.02 (p, ^3^*J* = 7.5 Hz, 2H; –CH_2_C*H*_2_CH_2_C(O)NHS); 1.61 (m, 603H, –CH_2_– of poly(εCL)); 1.34 (p, 302H, –CH_2_– of poly(εCL)) (poly(εCL)-**1**); δ: 7.34 (m, 5H, C_6_*H*_5_); 5.10 (s, 2H, PhC*H*_2_); 4.61 (s, 0.63H, –OC*H*_2_C(O)–); 4.05 (t, 291H, –OC*H*_2_– of poly(εCL)); 2.86 (s, 1.54H, –CH_2_– of NHS); 2.29 (t, 291H, C(O)CH_2_ of poly(εCL)); 1.63 (m, 592H, –CH_2_– of poly(εCL)); 1.37 (p, 297H, –CH_2_– of poly(εCL)) (poly(εCL)-**2**); δ: 7.34 (m, 5H, C_6_*H*_5_); 6.79 (s, 0.79H, –C*H* = of MI); 5.10 (s, 2H, PhC*H*_2_); 4.61 (s, 0.77H, –NC*H*_2_C(O)–); 4.04 (t, 298H, –OC*H*_2_– of poly(εCL)); 2.29 (t, 297H, C(O)CH_2_ of poly(εCL)); 1.64 (m, 600H, –CH_2_– of poly(εCL)); 1.37 (p, Hz, 303H, –CH_2_– of poly(εCL)) (poly(εCL)-**3**); δ: 7.33 (m, 5H, C_6_*H*_5_); 6.69 (s, 1.51H, –NC*H*_2_C(O)–); 5.09 (s, 2H, PhC*H*_2_); 4.04 (t, 315H, –OC*H*_2_– of poly(εCL)); 3.80 (t, ^3^*J* = 7.1 Hz, 1.51H, –NC*H*_2_CH_2_C(O)–); 2.61 (t, ^3^*J* = 7.1 Hz, 1.49H, –NCH_2_C*H*_2_C(O)–); 2.28 (t, 314H, C(O)CH_2_ of poly(εCL)); 1.62 (m, 634H, –CH_2_– of poly(εCL)); 1.35 (p, 314H, –CH_2_– of poly(εCL)) (poly(εCL)-**4**). ^1^H NMR spectra are presented in Figures S12–S15 in the Supplementary Materials.

#### 2.3.4. Polymerization of l-LA with Termination by **1** and **4**

A preheated 30 mL glass ampoule was equipped with a magnetic stir bar and a septum and then filled with dry argon. l-LA (3.00 g, 20.8 mmol, 120 eq.) was placed into the ampule, THF was added to total volume of 8.4 mL. The mixture was placed in ice bath, BHT-Mg (73 mg, 0.17 mmol Mg, 1 eq.) in THF solvent (2.0 mL) was added (resulting concentration of l-LA was 2 M). After 1 h of stirring, acyl chloride **1** or **4** (0.69 mmol, 4 eq.) in CH_2_Cl_2_ solvent (2 mL) was added. The mixture was allowed to warm to RT and stirred for 8 h. Reaction mixture was evaporated with CH_2_Cl_2_ twice for THF removing. The residue was dissolved in CH_2_Cl_2_ (40 mL), washed by 0.7 M aq. HCl, by water, dried over MgSO_4_ and filtered. The filtrate was evaporated to residual volume of 20 mL and poured into Et_2_O (100 mL). The polymer was filtered off, dried at 20 °C and 0.01 Torr. The yields were 2.82 (94%) and 2.94 g (98%) for poly(l-LA) functionalized by **1** and **4**, respectively. ^1^H NMR (CDCl_3_, 20 °C) δ: 7.35 (m, 5H, C_6_*H*_5_); 5.16 (q, ^3^*J* = 7.1 Hz, 257H, –OC*H*(CH_3_)– of poly(l-LA)); 2.83 (s, 4H, –CH_2_– of NHS); 2.73 (t, ^3^*J* = 7.5 Hz, 2H; –CH_2_CH_2_C*H*_2_C(O)NHS); 2.55 (t, ^3^*J* = 7.5 Hz, 2H; –C*H*_2_CH_2_CH_2_C(O)NHS); 2.09 (p, ^3^*J* = 7.5 Hz, 2H; –CH_2_C*H*_2_CH_2_C(O)NHS); 1.58 (d, ^3^*J* = 7.1 Hz, 771H, –CH_2_– of poly(l-LA)) (poly(l-LA)-**1**); δ: 7.34 (m, 5H, C_6_*H*_5_); 6.69 (s, 2H, –NC*H*_2_C(O)–); 5.16 (q, 312H, –OC*H*(CH_3_)– of poly(l-LA)); 3.84 (t, ^3^*J* = 7.1 Hz, 2H, –NC*H*_2_CH_2_C(O)–); 2.74 (t, ^3^*J* = 7.1 Hz, 2H, –NCH_2_C*H*_2_C(O)–); 1.58 (d, 975H, –CH_2_– of poly(l-LA)) (poly(l-LA)-**4**). ^1^H NMR spectra are presented in Figures S16 and S17 in the Supplementary Materials.

#### 2.3.5. Polymerization of EtEP with Termination by **1** and **4**

A preheated 30 mL glass ampoule was equipped with a magnetic stir bar and a septum and then filled with dry argon. EtEP (2.83 g, 20.8 mmol, 120 eq.) was placed into the ampule, CH_2_Cl_2_ was added to total volume of 8.4 mL. The mixture was placed in ice bath, cooled to 5 °C, BHT-Mg (73 mg, 0.17 mmol Mg, 1 eq.) in solvent THF (2.0 mL) was added (resulting concentration of EtEP was 2 M). After 1 h of stirring, acyl chloride **1** or **4** (0.69 mmol, 4 eq.) in CH_2_Cl_2_ solvent (2 mL) was added. The mixture was allowed to warm to RT and stirred for 4 h. Reaction mixture was precipitated into diethyl ether (100 mL) and centrifuged. The residue was dissolved in dry DMSO (8 mL) and precipitated into DMM (50 mL). The polymer was separated by decantation centrifugation and dried at 20 °C and 0.01 Torr. The yields were 1.50 (54%) and 1.65 g (59%) for poly(EtEP) functionalized by **1** and **4**, respectively. ^1^H NMR (CDCl_3_, 20 °C) δ: 7.35 (m, 5H, C_6_*H*_5_); 5.05 (d, ^3^*J*_HP_ = 9.0 Hz, 2H, PhC*H*_2_); 4.3–4.1 (m, 491H, –O(C*H*_2_)_2_O–); 2.82 (s, 4H, –CH_2_– of NHS); 2.69 (t, ^3^*J* = 7.4 Hz, 2H; –CH_2_CH_2_C*H*_2_C(O)NHS); 2.48 (t, ^3^*J* = 7.3 Hz, 2H; –C*H*_2_CH_2_CH_2_C(O)NHS); 2.03 (p, ^3^*J* = 7.3 Hz, 2H; –CH_2_C*H*_2_CH_2_C(O)NHS); 1.81 (m, 237H, –PC*H*_2_CH_3_); 1.15 (dt, ^3^*J* = 7.6 Hz, ^4^*J*_HP_ = 20.5 Hz, 360H, –PCH_2_C*H*_3_) (poly(EtEP)-**1**); δ: 7.36 (m, 5H, C_6_*H*_5_); 6.71 (s, 2H, –NC*H*_2_C(O)–); 5.06 (d, 2H, PhC*H*_2_); 4.3–4.1 (m, 521H, –O(C*H*_2_)_2_O–); 3.82 (t, ^3^*J* = 7.1 Hz, 2H, –NC*H*_2_CH_2_C(O)–); 2.66 (t, ^3^*J* = 7.1 Hz, 2H, –NCH_2_C*H*_2_C(O)–); 1.80 (m, 237H, –PC*H*_2_CH_3_); 1.16 (dt, 360H, –PCH_2_C*H*_3_) (poly(EtEP)-**4**). ^31^P NMR (CDCl_3_, 20 °C) δ: 35.3 (s). ^1^H NMR spectra are presented in Figures S18 and S19 in the Supplementary Materials.

#### 2.3.6. Synthesis of εCL/EtEP Block Copolymers with Termination by **1** and **4**

A preheated 30 mL glass ampoule was equipped with a magnetic stir bar and a septum and then with dry argon. εCL (2.30 mL, 2.37 g, 20.8 mmol, 120 eq.) was placed into the ampule, CH_2_Cl_2_ was added to total volume of 8.4 mL. The mixture was cooled to 5 °C, BHT-Mg (73 mg, 0.17 mmol Mg, 1 eq.) in solvent THF (2.0 mL) was added (resulting concentration of εCL was 2 M). After 4 h of stirring, EtEP (826 mg, 6.1 mmol, 35 eq.) was added. After 8 h of stirring, acyl chloride **1** or **4** (0.69 mmol, 4 eq.) in CH_2_Cl_2_ solvent (2 mL) was added. The mixture was allowed to warm to RT and stirred for 4 h. Reaction mixture was evaporated twice with CH_2_Cl_2_ for removal of THF. The residue was dissolved in CH_2_Cl_2_ (40 mL), washed by 0.7 M aq. HCl, by water, dried over MgSO_4_ and filtered. The filtrate was evaporated to residual volume of 20 mL and poured into Et_2_O (100 mL). The polymer was filtered off, dried at 20 °C and 0.01 Torr. The yields were 1.99 (62%) and 2.25 g (70%) for copolymers functionalized by **1** and **4**, respectively. ^1^H NMR (CDCl_3_, 20 °C) δ: 7.34 (m, 5H, C_6_*H*_5_); 5.10 (s, 2H, PhC*H*_2_); 4.3–4.1 (m, 53H, –O(C*H*_2_)_2_O–); 4.04 (t, 262H, –OC*H*_2_– of poly(εCL)); 2.83 (s, 4H, –CH_2_– of NHS); 2.71 (t, 2H; –CH_2_CH_2_C*H*_2_C(O)NHS); 2.51 (t, 2H; –C*H*_2_CH_2_CH_2_C(O)NHS); 2.29 (t, 259H, C(O)C*H*_2_ of poly(εCL)); 2.07 (p, 2H; –CH_2_C*H*_2_CH_2_C(O)NHS); 1.80 (m, 27H, –PC*H*_2_CH_3_); 1.63 (m, 528H, –CH_2_– of poly(εCL)); 1.36 (p, 265H, –CH_2_– of poly(εCL); 1.16 (dt, 43H, –PCH_2_C*H*_3_) (poly(εCL)-*b*-poly(EtEP)-**1**); δ: 7.34 (m, 5H, C_6_*H*_5_); 6.71 (s, 2H, –NC*H*_2_C(O)–); 5.10 (s, 2H, PhC*H*_2_); 4.3–4.1 (m, 80H, –O(C*H*_2_)_2_O–); 4.05 (t, 346H, –OC*H*_2_– of poly(εCL)); 3.83 (t, 2H, –NC*H*_2_CH_2_C(O)–); 2.67 (t, 2H, –NCH_2_C*H*_2_C(O)–); 2.29 (t, Hz, 344H, C(O)CH_2_ of poly(εCL)); 1.80 (m, 44H, –PC*H*_2_CH_3_); 1.64 (m, 698H, –CH_2_– of poly(εCL)); 1.35 (p, 348H, –CH_2_– of poly(εCL); 1.16 (dt, 62H, –PCH_2_C*H*_3_)) (poly(εCL)-*b*-poly(EtEP)-**4**). ^31^P NMR (CDCl_3_, 20 °C) δ: 35.3 (s). ^1^H NMR spectra are presented in Figures S20 and S21 in the Supplementary Materials.

#### 2.3.7. Polymerization of EtOEP with Termination by **1**, BHT-Mg Catalyst

A preheated 30 mL glass ampoule was equipped with a magnetic stir bar and a septum and then filled with dry argon. EtOEP (3.16 g, 20.8 mmol, 120 eq.) was placed into the ampule, CH_2_Cl_2_ was added to total volume of 8.4 mL. The mixture was placed in ice bath, cooled to 5 °C, BHT-Mg (73 mg, 0.17 mmol Mg, 1 eq.) in THF solvent (2.0 mL) was added (resulting concentration of EtOEP was 2 M). After 10 min of stirring, acyl chloride **1** (0.69 mmol, 4 eq.) in CH_2_Cl_2_ solvent (2 mL) was added. The mixture was allowed to warm to RT and stirred for 8 h. The reaction mixture was evaporated twice with CH_2_Cl_2_ for removal of THF. The residue was dissolved in CH_2_Cl_2_ (10 mL), precipitated into diethyl ether (80 mL). The sediment was dissolved in dry DMSO (10 mL) and precipitated into DMM (80 mL). The polymer was separated by centrifugation and dried at 20 °C and 0.01 Torr. The yield was 2.28 g (72%). ^1^H NMR (CDCl_3_, 20 °C) δ: 7.37 (m, 5H, C_6_*H*_5_); 5.07 (d, ^3^*J*_HP_ = 8.2 Hz, 2H, PhC*H*_2_); 4.25 (m, –O(C*H*_2_)_2_O–); 4.15 (m, –OC*H*_2_CH_3_) {894H}; 2.83 (s, 4H, –CH_2_– of NHS); 2.71 (t, ^3^*J* = 7.1 Hz, 2H; –CH_2_CH_2_C*H*_2_C(O)NHS); 2.44 (t, ^3^*J* = 7.2 Hz, 2H; –C*H*_2_CH_2_CH_2_C(O)NHS); 2.05 (p, ^3^*J* = 7.2 Hz, 2H; –CH_2_C*H*_2_CH_2_C(O)NHS); 1.34 (td, ^3^*J* = 6.9 Hz, ^5^*J*_HP_ = 1.1 Hz, 437H,–OCH_2_CH_3_). ^31^P NMR (CDCl_3_, 20 °C) δ: –1.20 (s). ^1^H NMR spectrum of poly(EtOEP)-**1** is presented in Figure S22 in the Supplementary Materials.

#### 2.3.8. Polymerization of EtOEP with Termination by **1**, TBD/BnOH Catalyst

A preheated 20 mL glass ampoule was equipped with a magnetic stir bar and a septum and then filled with dry argon. EtOEP (1.52 g, 10 mmol, 50 eq.) was placed into the ampule, CH_2_Cl_2_ was added to total volume of 3 mL. The catalyst solution was prepared in 5 mL glass vial by addition of BnOH (22 mg, 0.2 mmol) and TBD (28 mg, 0.2 mmol) to CH_2_Cl_2_ (1 mL). The first solution was cooled to 5 °C, the catalyst solution was injected by a syringe, and CH_2_Cl_2_ was added to total volume of 5 mL (2 M concentration of EtOEP). After 30 min of stirring, acyl chloride **1** (1.2 mmol, 6 eq.) in CH_2_Cl_2_ solvent (1 mL) was added. The mixture was allowed to warm to RT and stirred for 8 h. The reaction mixture was evaporated to residual volume of 3 mL, and precipitated into diethyl ether (20 mL). The residue was dried at 20 °C and 0.01 Torr. ^1^H NMR spectrum is presented in Figure S23 in the Supplementary Materials.

#### 2.3.9. Reactions of poly(εCL) and poly(EtOEP) with **1**

The samples of poly(εCL) and poly(EtOEP) were prepared by the methods described in Section 2.3.3 and Section 2.3.7, respectively, polymerization was terminated by the addition of 5 eq. AcOH. A total of 1 g of polymer was dissolved in CH_2_Cl_2_ (10 mL), and acyl chloride **1** (6 eq.) was added. After 8 h of stirring, the solution was poured into Et_2_O (50 mL), precipitated polymer was filtered off, dried in vacuo and analyzed using SEC and NMR spectroscopy. ^1^H NMR spectra of the starting and NHS-functionalized poly(εCL) and poly(EtOEP) are presented in Figures S24 and S25, respectively, in the Supplementary Materials.

### 2.4. Reactions of NHS-Functionalized Polymers with ^i^BuNH_2_

#### 2.4.1. Reactions in CH_2_Cl_2_, Synthesis of poly(εCL)-1-N and poly(l-LA)-1-N

According to previously reported procedure [65], NHS-functionalized polymer (200 mg) was dissolved in CH_2_Cl_2_ (2 mL) and treated with ^i^BuNH_2_ (4 equivalents relative to amount of NHS groups in the polymer). After 6 h of stirring at RT, CH_2_Cl_2_ (8 mL) was added, organic phase was washed by 1M aq. HCl, by water, dried over MgSO_4_ and filtered. The filtrate was evaporated to residual volume of 4 mL, poured into Et_2_O (50 mL), filtered off and dried at 20 °C and 0.01 Torr. The yields were 120 mg (60%) for poly(εCL)-**1**-*N*, 154 mg (77%) for poly(l-LA)-**1**-*N*.

#### 2.4.2. Reactions in Aqueous Media, Synthesis of poly(EtEP)-1-N and poly(EtOEP)-1-N

Taking into account previously reported procedures [66,67], NHS-functionalized polymer (100 mg) was dissolved in water (1 mL) and treated with ^i^BuNH_2_ (4 equivalents relative to amount of NHS groups in the polymer). After 6 h at RT, the reaction solution was dialyzed in distilled water using benzoylated cellulose dialysis membrane D2272 (MWCO 2000). The solution of the polymer was lyophilized, the residue was dissolved in CH_2_Cl_2_ (2 mL) and precipitated into DMM (20 mL). The polymer was separated by centrifugation and dried at 20 °C and 0.01 Torr. The yields were 54 mg (54%) for poly(EtEP)-**1**-*N*, 59 mg (59%) for poly(EtOEP)-**1**-*N*.

#### 2.4.3. NMR Data

Poly(εCL)-**1**-*N*: ^1^H NMR (CDCl_3_, 20 °C) δ: 7.34 (m, 5H, C_6_*H*_5_); 5.10 (s, 2H, PhC*H*_2_); 4.05 (t, 261H, –OC*H*_2_– of poly(εCL)); 3.07 (t, 1.4H, >NHC*H*_2_CH<); 2.30 (t, 260H, C(O)C*H*_2_ of poly(εCL)); 2.23 (t, ^3^*J* = 6.6 Hz, 1.5H; –C*H*_2_CH_2_CH_2_C(O)NH^i^Bu); 1.64 (m, 525H, –CH_2_– of poly(εCL)); 1.37 (p, 263H, –CH_2_– of poly(εCL); 0.89 (d, ^3^*J* = 6.8 Hz, 4.5H, CH(C*H*_3_)_2_). See Figure S26 in the Supplementary Materials.

Poly(l-LA)-**1**-*N*: ^1^H NMR (CDCl_3_, 20 °C) δ: 7.34 (m, 5H, C_6_*H*_5_); 6.03 (bt, 1H, -N*H*-); 5.15 (q, 206H, –OC*H*(CH_3_)–); 3.06 (t, 2H, >NHC*H*_2_CH<); 2.44 (m, 2H, –CH_2_CH_2_C*H*_2_C(O)NH^i^Bu); 2.24 (m, 2H, –C*H*_2_CH_2_CH_2_C(O)NH^i^Bu); 2.01 (p, 2H, –CH_2_C*H*_2_CH_2_C(O)NH^i^Bu); 1.57 (d, 618H, –OCH(C*H*_3_)–); 0.90 (d, 6H, CH(C*H*_3_)_2_). See Figure S27 in the Supplementary Materials.

Poly(EtEP)-**1**-*N*: ^1^H NMR (CDCl_3_, 20 °C) δ: 7.35 (m, 5H, C_6_*H*_5_); 5.06 (d, 2H, PhC*H*_2_); 4.3–4.1 (m, 710H, –O(C*H*_2_)_2_O–); 3.02 (t, 1.8H, >NHC*H*_2_CH<); 2.39 (t, 2H, –CH_2_CH_2_C*H*_2_C(O)NH^i^Bu); 2.24 (t, 2H, –C*H*_2_CH_2_CH_2_C(O)NH^i^Bu); 1.94 (p, 2H, –CH_2_C*H*_2_CH_2_C(O)NH^i^Bu); 1.80 (m, 359H, –PC*H*_2_CH_3_); 1.16 (dt, 537H, –PCH_2_C*H*_3_). ^31^P NMR (CDCl_3_, 20 °C) δ: 35.32. See Figures S28 and S29 in the Supplementary Materials.

Poly(EtOEP)-**1**-*N*: ^1^H NMR (CDCl_3_, 20 °C) δ: 7.35 (m, 5H, C_6_*H*_5_); 5.04 (d, 2H, PhC*H*_2_); 4.21 (m, –O(C*H*_2_)_2_O–); 4.12 (m, –OC*H*_2_CH_3_) {934H}; 3.00 (d and d, 0.8H, >NC*H*_2_CH<); 2.37 (t, ^3^*J* = 6.6 Hz, 0.98H; –C*H*_2_CH_2_CH_2_C(O)NH^i^Bu); 2.21 (t, ^3^*J* = 6.6 Hz, 0.8H; –CH_2_CH_2_C*H*_2_C(O)NH^i^Bu); 1.93 (p, ^3^*J* = 6.6 Hz, 0.8H; –CH_2_C*H*_2_CH_2_C(O)NH^i^Bu); 1.71 (m, 0.4H, >NCH_2_C*H*<); 1.31 (td, ^3^*J* = 6.9 Hz, ^5^*J*_HP_ = 1.1 Hz, 458H,–OCH_2_CH_3_); 0.85 (d, ^3^*J* = 6.8 Hz, 2.6H, CH(C*H*_3_)_2_). ^31^P NMR (CDCl_3_, 20 °C) δ: –1.21. See Figures S30 and S31 in the Supplementary Materials.

### 2.5. Reactions of MI-Functionalized Polymers with HSCH_2_COOMe

#### 2.5.1. Reactions in Organic Solvent, Synthesis of poly(εCL)-4-S and poly(l-LA)-4-S

According to previously reported procedure [68,69], MI-functionalized polymer (200 mg) was dissolved in CH_2_Cl_2_ (2 mL) and treated with HSCH_2_COOMe and Et_3_N (2 and 4 eqs., respectively, relative to amount of MI groups in the polymer). After 6 h at RT, CH_2_Cl_2_ (8 mL) was added, organic phase was washed by 1M aq. HCl, by water, dried over MgSO_4_ and filtered. The filtrate was evaporated under reduced pressure to residual volume of 4 mL and poured into Et_2_O (50 mL). The polymer was filtered off, dried at 20 °C and 0.01 Torr. The yields were 125 mg (62%) for poly(εCL)-**4**-*S* and 120 mg (60%) for poly(l-LA)-**4**-*S*.

#### 2.5.2. Reaction in Aqueous Media, Synthesis of poly(EtEP)-**4**-*S*

Analogous to the previously reported procedure [70], poly(EtEP)-**4** (100 mg, 0.005 mmol of MI groups) was dissolved in H_2_O (1 mL) and treated with HSCH_2_COOMe (0.9 µL, 2 eq.). After 6 h of stirring at RT, the reaction solution was dialyzed in distilled water using benzoylated cellulose dialysis membrane D2272 (MWCO 2000). The solution of the polymer was lyophilized, the residue was dissolved in CH_2_Cl_2_ (2 mL) and precipitated into DMM (20 mL). The polymer was separated by centrifugation and dried at 20 °C and 0.01 Torr. The yield was 69 mg (69%).

#### 2.5.3. NMR Data

Poly(εCL)-**4**-S: ^1^H NMR (CDCl_3_, 20 °C) δ: 7.34 (m, 5H, C_6_*H*_5_); 5.10 (s, 2H, PhC*H*_2_); 4.04 (t, 241H, –OC*H*_2_– of poly(εCL)); 3.89 & 3.37 (d, d, ^2^*J* = 15.8 Hz, 1.5H, –SC*H*_2_–); 3.80 (t, ^3^*J* = 7.1 Hz, 1.5H, –NC*H*_2_CH_2_C(O)–); 3.75 (s, ~2H, –SC*H*_3_); 3.15 (dd, ^2^*J* = 18.9 Hz, ^3^*J* = 9.0 Hz, –CH(S)C*H*_2_–); 2.60 (t, ^3^*J* = 7.1 Hz, 1.5H, –NCH_2_C*H*_2_C(O)–); 2.51 (dd, ^2^*J* = 18.9 Hz, ^3^*J* = 3.8 Hz, –CH(S)C*H*_2_–); 2.30 (t, 242H, C(O)CH_2_ of poly(εCL)); 1.64 (m, 487H, –CH_2_– of poly(εCL)); 1.37 (p, 241H, –CH_2_– of poly(εCL)).

Poly(l-LA)-**4**-S: ^1^H NMR (CDCl_3_, 20 °C) δ: 7.35 (m, 5H, C_6_*H*_5_); 5.15 (q, 275H, –OC*H*(CH_3_)–); 4.02 (m, 1H, –CH_2_C*H*S–); 3.89 (d, ^2^*J* = 15.0 Hz, 1H, –SC*H*_2_–); 3.84 (m, 2H, –NC*H*_2_CH_2_C(O)–); 3.76 (s, 3H, –OC*H*_3_); 3.37 (d, ^2^*J* = 15.0 Hz, 1H, –SC*H*_2_–); 3.15 (dd, 1H, –C*H*_2_CHS–); 2.76 (m, 2H, –NCH_2_C*H*_2_C(O)–); 2.49 (dd, 1H, –C*H*_2_CHS–); 1.57 (d, 832H, –OCH(C*H*_3_)–).

Poly(EtEP)-**4**-S: ^1^H NMR (CDCl_3_, 20 °C) δ: 7.36 (m, 5H, C_6_*H*_5_); 5.06 (d, 2H, PhC*H*_2_); 4.3–4.1 (m, 508H, –O(C*H*_2_)_2_O–); 3.86 (d, ^2^*J* = 15.9 Hz, 1H, –SC*H*_2_–); 3.80 (t, ^3^*J* = 7.1 Hz, 2H, –NC*H*_2_CH_2_C(O)–); 3.74 (s, 3H, –OC*H*_3_); 3.37 (d, ^2^*J* = 15.9 Hz, 1H, –SC*H*_2_–); 2.65 (t, ^3^*J* = 7.1 Hz, 2H, –NCH_2_C*H*_2_C(O)–); 1.80 (m, 250H, –PC*H*_2_CH_3_); 1.16 (dt, 377H, –PCH_2_C*H*_3_). ^31^P NMR (CDCl_3_, 20 °C) δ: 35.3 (s). ^31^P NMR (CDCl_3_, 20 °C) δ: 35.25.

## 3. Results and Discussion

### 3.1. Functionalization of the Polymers by Termination of Living Polymerization

The synthesis of the functionalized polymers by termination of “living” ROP was performed previously using both organocatalyst DBU [37,44] and Al-based coordination catalyst [45,46]. However, degree of polymerization (DP_n_) in these experiments stood at 30–40, the polymers obtained were not considered as a base for functionalized polymer materials having required strength and plastic characteristics. We proposed that the synthesis of high molecular weight (MW) functionalized polymers can be based on the use of the complexes of more active metal than Al. Among the coordination catalysts available, derivatives on nontoxic metals Al, Zn, Ca and Mg [55,71,72], we chose the most active magnesium catalyst BHT-Mg (Scheme 2b) that had been previously used in ROP of lactones, lactides, cyclic phosphonates, and phosphates [47,49,50,52,54,73]. The choice of the magnesium catalyst was also due to high reactivity of magnesium alkoxides in reactions with acyl chlorides, demonstrated previously in the few publications [74,75].

Using BHT-Mg as a single-component catalyst, we studied living ROP of εCL, followed by the termination with different reagents. Addition of DSC to the reaction mixture resulted in a formation of NHS-substituted poly(εCL), however, degree of functionalization was only 33% (Table 1, Entry 1 and Figure S9 in the Supplementary Materials). The addition of SA, followed by the reaction with *N*-hydroxysuccinimide/DCC, leads to the NHS-substituted polymer with degree of functionalization of 51% (Table 1, Entry 2 and Figure S10 in the Supplementary Materials).

We assumed that acyl chlorides could be more efficient reagents for the functionalization of polymers via termination of BHT-Mg catalyzed ROP. In the preliminary experiment, we polymerized l-LA in the presence of BHT-Mg at 80:1 l-LA/Mg ratio, and terminated the reaction by the addition of four equivalents of AcCl. Acetyl-terminated poly(l-LA) was isolated, degree of functionalization was ~100% (Figure S11 in the Supplementary Materials). Similar results had been obtained in AcCl-terminated ROP of εCL. When NHS-substituted acyl chloride **1** (Scheme 2b) [61] was used in **1**/Mg ratio of 4:1, the near quantitative degree of the functionalization of poly(εCL) was achieved (Table 1, Entry 3 and Figure S12 in the Supplementary Materials).

In view of the results of these experiments, we proposed that termination of BHT-Mg catalyzed coordination ROP by functionalized acyl chlorides may be the most effective method for the obtaining of NHS- and MI-substituted polymers. In addition to **1**, we synthesized NHS-derived acyl chloride **2** (new compound) and MI-derived acyl chlorides **3 [63]** and **4** [64] (Scheme 2b). When **2** was used for the termination of εCL ROP, the degree of functionalization was 39% (Table 1, Entry 4 and Figure S13 in the Supplementary Materials). Low reactivity of **2** may be due to the formation of *k*^2^-chelate by carbonyl and ether oxygen atoms in **2** and BHT-Mg species.

MI-substituted acyl chlorides **3** and **4** demonstrated high reactivities, but the degrees of functionalization were far from quantitative in both cases (Figures S14 and S15 in the Supplementary Materials). The yield of MI-substituted polymer was substantially higher when **4** was used (Table 1, Entries 5 and 6). In the light of the results obtained, acyl chlorides **1** and **4** were selected for termination/functionalization of the ROP of other cyclic substrates.

**Figure 1:**
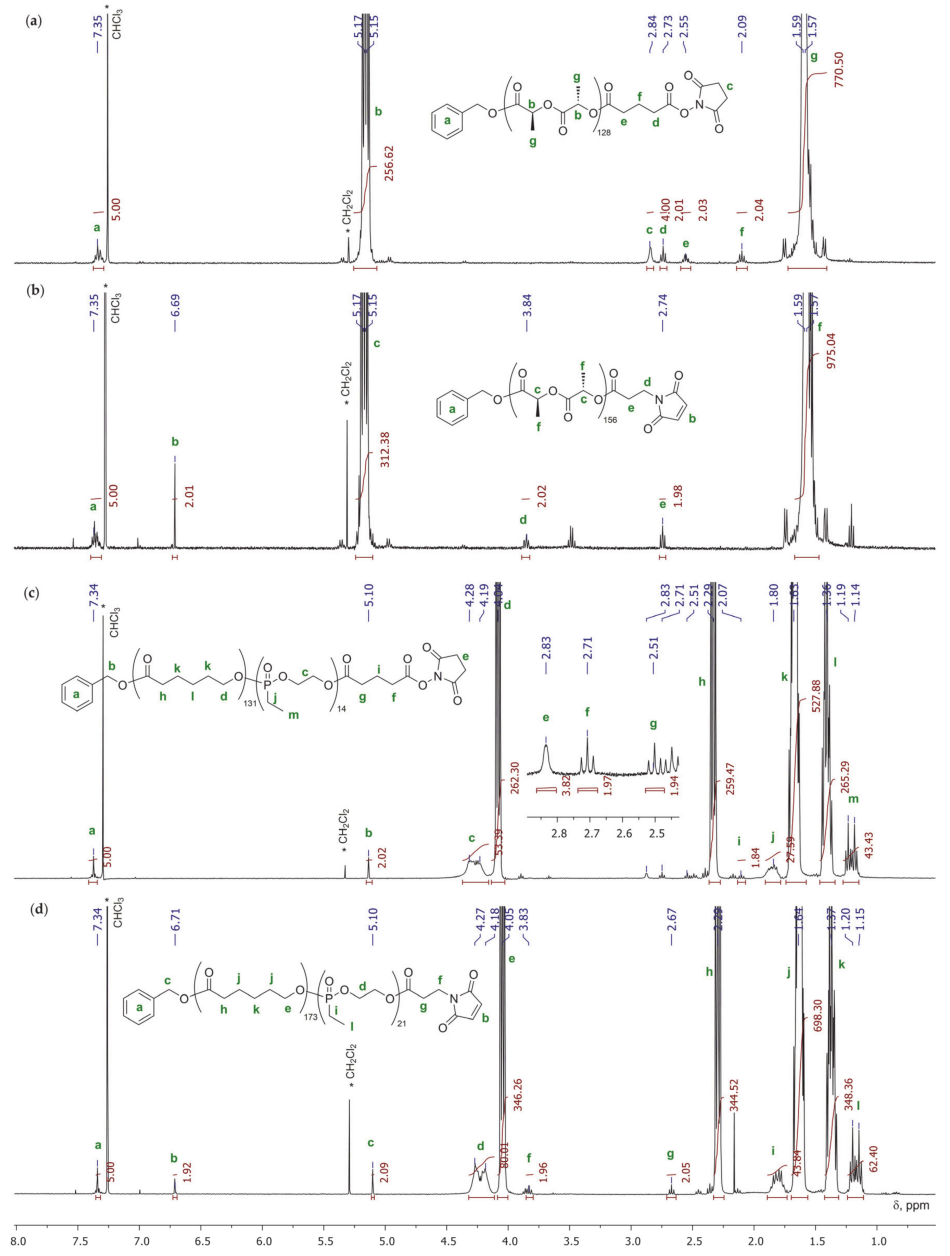
^1^H NMR spectra of NHS- and MI-functionalized polymers (Table 1, Entries 7 (**a**), 8 (**b**), 11 (**c**), and 12 (**d**)). The signals of the protons of the fragments of functionalized polymers are marked as letters (bold and green) the solvent impurities are marked by asterisks (*). 3.2. Functionalization by the Reaction of the Polymers with Acyl Chloride **1**.

When BHT-Mg catalyzed ROP of l-LA was terminated by acyl chlorides **1** and **4**, the degrees of functionalization were found to be almost quantitative (Table 1, Entries 7 and 8 and Figure 1). The experiments with termination of the ROP of EtEP by **1** and **4** were also successful (Table 1, Entries 9 and 10, Figures S18 and S19 in the Supplementary Materials). In the synthesis of poly(εCL)-*b*-poly(EtEP) copolymers termination of the “living” polymer chain by **1** and **4** resulted in the obtaining of exhaustively NHS- and MI-functionalized copolymers with expected comonomer ratios (Table 1, Entries 11 and 12, Figure 1). Termination of EtOEP ROP by **1** resulted in poly(EtOEP) with high degree of functionalization (Table 1, Entry 13 and Figure S22 in the Supplementary Materials).

In the final experiment (Table 1, Entry 14), we initiated polymerization of EtOEP by TBD/BnOH, and added 4 eq. of acyl chloride **1** after 30 min of the reaction. The obtained polymer had degree of the functionalization less than 50%. Perhaps more significantly, ^1^H NMR spectra of reprecipitated polymer mainly contained the signals of the side products with (CH_2_)_3_C(O)NHS fragments (see Figure S23 in the Supplementary Materials), and SEC data pointed to partial fragmentation of the polymer.

### 3.2. Functionalization by the Reaction of the Polymers with Acyl Chloride **1**

In this way, it is BHT-Mg catalyst that provides termination of the ROP of lactones, lactides, ethylene phosphonates, and ethylene phosphates by acyl chlorides with high degree of functionalization without the degradation of the polymer backbone.

The obvious alternative for the termination of “living” BHT-Mg catalyzed polymerization by acyl chlorides is a reaction of isolated polymers that contain –OH end groups with the same acyl chlorides. We synthesized the sample of poly(εCL) with degree of polymerization (DP_n_) ~125, and carried out the reaction of this polymer with acyl chloride **1** in CH_2_Cl_2_. NHS-functionalized polymer was obtained (see Section 2.3.8), the ratio of BnO initiator group and NHS end group in this polymer was 1:0.76 (Figure S24 in the Supplementary Materials). As a result of the same experiment with poly(EtOEP) (DP_n_ ~210, see Section 2.3.8), the degree of functionalization was only 0.55 (Figure S25 in the Supplementary Materials). Thus, the two-stage approach, based on functionalization of –OH end groups of the separated polymers by acyl chlorides, was substantially inferior to the method, based on the reaction of the active polymeryl-Mg-BHT complex with acyl chlorides.

### 3.3. Reactions of NHS-Functionalized Polymers with Amine-Containing Compounds

**Scheme 3:**
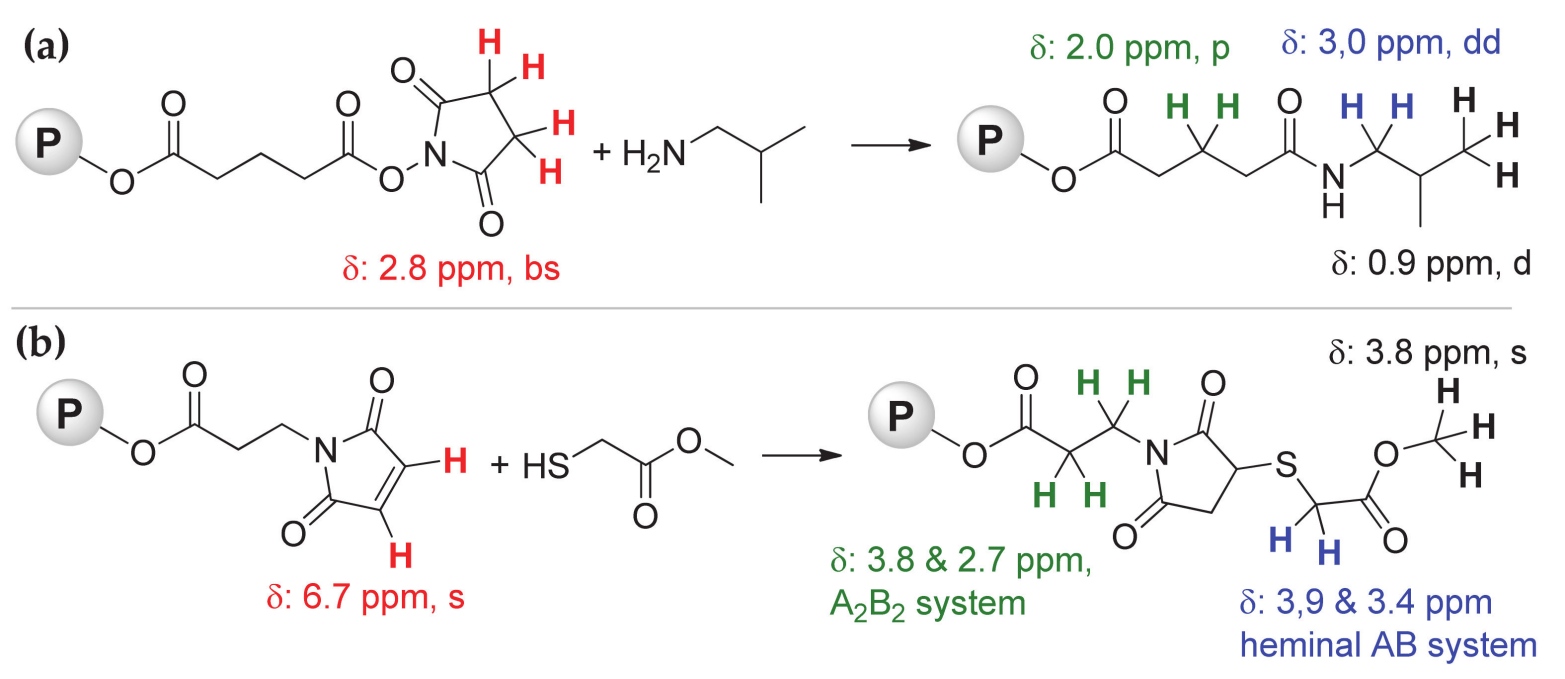
Reactions of NHS- and MI-functionalized polymers with model amine and thiol, ^1^H NMR criteria of the completion of the processes for NHS- and MI-terminated polymers, (**a**) and (**b**), respectively.

The reactions of NHS-substituted organic compounds with amines are widely presented in the chemical literature, these reactions can be conducted under various conditions using both organic solvents and water media. Among known methods, we chose reactions in CH_2_Cl_2_ [65,69] and in water [66,67]. Other common solvents, DMF and DMSO, in our view, are less applicable for polymer modification owing to the complexity of the further removal of these solvents. In our experiments, we studied interaction of NHS-modified polymers, derivatives of **1**, with ^i^BuNH_2_. The analysis of ^1^H NMR spectra was performed with account of the characteristic signals of the starting NHS derivatives and isobutylamide fragment of the product (Scheme 3a). Degrees of functionalization were calculated from the ratios of the integral intensities of these characteristic signals and signals of BnO group.

In the reaction of poly(εCL)-**1** with ^i^BuNH_2_ in CH_2_Cl_2_ corresponding amide was obtained with total degree of amide functionalization ~0.7. Given the low accuracy of chain-end group integration, εCL/end groups ratio in the product poly(εCL)-**1**-N (Figure S26 in the Supplementary Materials) was in line with the ratio of εCL fragments, BnO and NHS groups in the starting polymer. The same result was achieved in the reaction of poly(l-LA)-**1** with ^i^BuNH_2_. Characteristic multiplicity of the signals of –OC(O)C*H*_2_– in poly(l-LA)-**1**-N (Figure S27 in the Supplementary Materials) can be attributed to chirality of l-lactate fragment. These signals can be used in ^1^H NMR spectral identification of poly(l-LA) covalent binding with biomedically significant amines when using NHS functionalization of poly(l-LA) by acyl chloride **1**.

The reactions of water-soluble poly(EtEP)-**1** and poly(EtOEP)-**1** were conducted in aqueous media, purification of the substituted polymers from low MW impurities was made using dialysis of the polymer solutions. Degrees of amide functionalization were of the values of 0.7 and 0.4 for poly(EtEP)-**1** and poly(EtOEP)-**1**, respectively, in relation to BnO fragment (Figures S28 and S30 in the Supplementary Materials). In this way, this approach is of limited effectiveness for covalent binding of NHS-functionalized poly(ethylene phosphate)s, obtained by termination of the ROP by acyl chloride **1**.

### 3.4. Reactions of MI-Functionalized Polymers with Thiol-Containing Compounds

Reaction of MI-functionalized compounds with thiols [18,21] can be conducted in both organic [65] and aqueous [66,67] media similar to the reactions of NHS-functionalized compounds with amines. In our experiments, we chose HSCH_2_COOMe as a model thiol, the assignment of the signals in ^1^H NMR spectra (Scheme 3b) was based on the previously reported data [76,77].

The reactions of poly(εCL)-**4** and poly(l-LA)-**4** with HSCH_2_COOMe were conducted in CH_2_Cl_2_. In both examples, the degree of thiol binding can be estimated as ~0.8 (Figures S32 and S33 in the Supplementary Materials). Even higher degree of thiol binding was reached for poly(EtEP)-**4** in aqueous media (Figure S34 in the Supplementary Materials). This result is highly promising for the development of polymer–protein conjugates by the reaction of cysteine fragments in protein with MI-functionalized hydrophilic and biodegradable poly(ethylene phosphonate)s.

## 4. Conclusions

Termination of BHT-Mg catalyzed “living” ROP by functionalized acyl chlorides allowed to obtain NHS- and MI-containing polymers for different types of cyclic substrates: εCL, l-LA, EtEP and EtOEP. When using of acyl chloride **1** the degree of NHS functionalization was almost quantitative for poly(εCL), poly(l-LA), poly(EtEP) and poly(EtOEP). The results of the use of acyl chloride **4** were less successful for MI functionalization of poly(εCL), however, MI-terminated poly(l-LA) and poly(EtEP) were synthesized with almost quantitative yields.

Alternative approaches to functionalization, based on termination of BHT-Mg catalyzed ROP by conventional reagents (DSC or SA/*N*-hydroxysuccinimide/DCC), termination of TBD-catalyzed ROP by acyl chloride and on the reactions of –OH end-group containing polymers with acyl chloride, were not sufficiently effective.

The ability of NHS- and MI-functionalized polymers to further binding with amines and thiols without degradation of the polymer backbone was confirmed experimentally in model reactions with ^i^BuNH_2_ and HSCH_2_COOMe, respectively. These reactions were conducted in organic solvent (CH_2_Cl_2_) and in aqueous media. End-group analysis of the ^1^H NMR spectra showed no marked difference in comonomer/end group ratios for starting functionalized polymers and products of their further modification. Note that such analysis for the broad spectrum of functionalized polymers was performed for the first time ever.

In this way, suggested synthetic approach to NHS- and MI-functionalized polymers allows to synthesize high molecular weight products (DPn = 110–170), based on lactones, lactides, cyclic phosphonates, and cyclic phosphates, that are able to bind with amines and thiols, respectively. Relatively high MW values allow to consider these functionalized polymers as a base of chemically active polymer articles with a broad perspective of biomedical applications.

## Figures and Tables

**Table 1:** Synthesis of NHS- and MI-functionalized polymers via termination of BHT-Mg catalyzed “living” ROP by DSC, SA and acyl chlorides **1**–**4**.

Entry	Mon1	Mon2	ROP Termination Agent (TA)	(Mon1)/(Mon2)/(Cat)/(TA) Ratio	Mon1 conv., % ^1^	Mon2 conv., % ^1^	*M*_n_·10^3^ (theor) ^1^	*M*_n_·10^3^ (NMR) ^2^	*M*_n_·10^3^ (SEC) ^3^	*Đ*_M_ (SEC) ^3^	Comp. of Polymer: Mon1/Mon2/FG (NMR)
1	εCL	-	DSC	50/-/1/5	>99	-	5.96	6.0	6.4	1.19	50/-/0.33
2	εCL	-	SA, NHS/DCC	50/-/1/5	>99	-	5.96	7.0	7.4	1.22	59/-/0.51
3	εCL	-	**1**	120/-/1/4	>99	-	14.02	17.3	16.1	1.18	149/-/1.0
4	εCL	-	**2**	120/-/1/4	>99	-	14.02	16.9	18.2	1.29	145/-/0.39
5	εCL	-	**3**	120/-/1/4	>99	-	13.96	17.3	16.5	1.23	149/-/0.40
6	εCL	-	**4**	120/-/1/4	>99	-	13.94	18.2	22.4	1.15	157/-/0.76
7	l-LA	-	**1**	120/-/1/4	>99	-	17.62	18.8	21.7	1.43	128/-/1.0
8	l-LA	-	**4**	120/-/1/4	>99	-	17.56	22.8	25.6	1.55	156/-/1.0
9	EtEP	-	**1**	120/-/1/4	94	-	15.67	17.1	14.3	1.19	123/-/1.0
10	EtEP	-	**4**	120/-/1/4	95	-	15.78	17.9	14.5	1.18	130/-/1.0
11	εCL	EtEP	**1**	120/35/1/4	>99	65	17.11	17.1	21.4	1.48	131/14/1.0
12	εCL	EtEP	**4**	120/35/1/4	>99	79	17.72	22.9	19.5	1.46	173/21/1.0
13	EtOEP	-	**1**	120/-/1/4	96	-	17.84	22.5	16.5	1.48	146/-/1.0
14 ^4^	EtOEP	-	**1**	50/-/1/4	>99	-	7.85	8.3	5.2	1.86	53/-/<0.5

^1^ Determined by the analysis of ^1^H NMR spectra of the reaction mixtures. ^2^ Determined by the ratio of the integral intensities of the signals of polymer and BnO initiator in ^1^H NMR spectra. ^3^ Determined by SEC in THF vs. polystyrene standards for poly(*ε*CL) and poly(l-LA) (Entries 1–8, 11, 12), or in DMF vs. poly(ethylene glycol) standards for poly(EtEP) and poly(EtOEP) (Entries 9, 10, 13). ^4^ TBD/BnOH (1:1) as a catalyst.

## Data Availability

The data presented in this study are available on request from the corresponding author.

## Supplementary Materials

The following are available online at https://www.mdpi.com/2073-4360/13/6/868/s1, Figure S1: ^1^H NMR spectrum (400 MHz, CDCl_3_, 20 °C) of the acyl chloride **1**, Figure S2: ^13^C NMR spectrum (101 MHz, CDCl_3_, 20 °C) of the acyl chloride **1**, Figure S3: ^1^H NMR spectrum (400 MHz, CDCl_3_, 20 °C) of the acyl chloride **2**, Figure S4: ^13^C NMR spectrum (101 MHz, CDCl_3_, 20 °C) of the acyl chloride **2**, Figure S5: ^1^H NMR spectrum (400 MHz, CDCl_3_, 20 °C) of the acyl chloride **3**, Figure S6: ^13^C NMR spectrum (101 MHz, CDCl_3_, 20 °C) of the acyl chloride **3**, Figure S7: ^1^H NMR spectrum (400 MHz, CDCl_3_, 20 °C) of the acyl chloride **4**, Figure S8: ^13^C NMR spectrum (101 MHz, CDCl_3_, 20 °C) of the acyl chloride **4**, Figure S9: ^1^H NMR spectrum (400 MHz, CDCl_3_, 20 °C) of NHS-terminated poly(εCL) (Table 1, Entry 1), Figure S10: ^1^H NMR spectrum (400 MHz, DMSO-d_6_, 20 °C) of SA/NHS-terminated poly(εCL) (Table 1, Entry 2), Figure S11: ^1^H NMR spectrum (400 MHz, CDCl_3_, 20 °C) of acetyl-terminated poly(l-LA). Figure S12: ^1^H NMR spectrum (400 MHz, CDCl_3_, 20 °C) of poly(εCL)-**1** (Table 1, Entry 3), Figure S13: ^1^H NMR spectrum (400 MHz, CDCl_3_, 20 °C) of poly(εCL)-**2** (Table 1, Entry 4), Figure S14: ^1^H NMR spectrum (400 MHz, CDCl_3_, 20 °C) of poly(εCL)-**3** (Table 1, Entry 5), Figure S15: ^1^H NMR spectrum (400 MHz, CDCl_3_, 20 °C) of poly(εCL)-**4** (Table 1, Entry 6), Figure S16: ^1^H NMR spectrum (400 MHz, CDCl_3_, 20 °C) of poly(l-LA)-**1** (Table 1, Entry 7), Figure S17: ^1^H NMR spectrum (400 MHz, CDCl_3_, 20 °C) of poly(l-LA)-**4** (Table 1, Entry 8), Figure S18: ^1^H NMR spectrum (400 MHz, CDCl_3_, 20 °C) of poly(EtEP)-**1** (Table 1, Entry 9), Figure S19: ^1^H NMR spectrum (400 MHz, CDCl_3_, 20 °C) of poly(EtEP)-**4** (Table 1, Entry 10), Figure S20: ^1^H NMR spectrum (400 MHz, CDCl_3_, 20 °C) of poly(εCL)-*b*-poly(EtEP)-**1** (Table 1, Entry 11), Figure S21: ^1^H NMR spectrum (400 MHz, CDCl_3_, 20 °C) of poly(εCL)-*b*-poly(EtEP)-**4** (Table 1, Entry 12), Figure S22: ^1^H NMR spectrum (400 MHz, CDCl_3_, 20 °C) of poly(EtOEP)-**1** (Table 1, Entry 13), Figure S23: ^1^H NMR spectrum (400 MHz, CDCl_3_, 20 °C) of poly(EtOEP)-**1** obtained using TBD/BnOH initiation, Figure S24: ^1^H NMR spectrum (400 MHz, CDCl_3_, 20 °C) of NHS-functionalized poly(εCL) obtained by the reaction of acyl chloride **1** with poly(εCL), Figure S25: ^1^H NMR spectrum (400 MHz, CDCl_3_, 20 °C) of NHS-functionalized poly(EtOEP) obtained by the reaction of acyl chloride **1** with poly(EtOEP). Figure S26: ^1^H NMR spectrum (400 MHz, CDCl_3_, 20 °C) of poly(εCL)-**1**-N, Figure S27: ^1^H NMR spectrum (400 MHz, CDCl_3_, 20 °C) of poly(l-LA)-**1**-N, Figure S28: ^1^H NMR spectrum (400 MHz, CDCl_3_, 20 °C) of poly(EtEP)-**1**-N, Figure S29: ^31^P NMR spectrum (162 MHz, CDCl_3_, 20 °C) of poly(EtEP)-**1**-N, Figure S30: ^1^H NMR spectrum (400 MHz, CDCl_3_, 20 °C) of poly(EtOEP)-**1**-N, Figure S31: ^31^P NMR spectrum (162 MHz, CDCl_3_, 20 °C) of poly(EtOEP)-**1**-N, Figure S32: ^1^H NMR spectrum (400 MHz, CDCl_3_, 20 °C) of poly(εCL)-**4**-S, Figure S33: ^1^H NMR spectrum (400 MHz, CDCl_3_, 20 °C) of poly(l-LA)-**4**-S, Figure S34: ^1^H NMR spectrum (400 MHz, CDCl_3_, 20 °C) of poly(EtEP)-**4**-S, Figure S35: ^31^P NMR spectrum (162 MHz, CDCl_3_, 20 °C) of poly(EtEP)-**4**-S.
