# Supplementary material for: Functionalized Biodegradable Polymers via Termination of Ring-Opening Polymerization by Acyl Chlorides
Source: Polymers (Basel). 2021 Mar 11;13(6):868. doi: 10.3390/polym13060868 (PMC8002085; doi:10.3390/polym13060868)
Supplement: Supplementary file 1 [file polymers-13-00868-s001.pdf]

Functionalized Biodegradable Polymers via Termination of Ring-Opening Polymerization by Acyl Chlorides

Ilya Nifant'ev [1,2,3,*], Andrey Shlyakhtin [1], Vladimir Bagrov [1], Evgeny Shaputkin [1], Alexander Tavtorkin [2] and Pavel Ivchenko [1,2]

[1] Chemistry Department, M.V. Lomonosov Moscow State University, 1–3 Leninskie Gory, 119991 Moscow, Russian Federation; inif@org.chem.msu.ru (I.N.); shlyahtinav@mail.ru (A.S.); vlabag@yandex.ru (V.B.); evgeny.shaputkin@yandex.ru (E.S.); phpasha1@yandex.ru (P.I.).
[2] Laboratory of Organometallic Catalysis, A.V. Topchiev Institute of Petrochemical Synthesis RAS, 29 Leninsky Pr., 119991 Moscow, Russian Federation; tavtorkin@yandex.ru (A.T.).
[3] Faculty of Chemistry, National Research University Higher School of Economics, 20 Miasnitskaya Str., Moscow 101000, Russian Federation.
* Correspondence: ilnif@yahoo.com; Tel.: +7-495-939-4098

Supplementary Information

S1. Synthesis of NHS- and MI-functionalized acyl chlorides 2–5

S2. Synthesis of functionalized polymers 6–14

S3. Reactions of functionalized polymers with $^iBuNH_2$ and $HSCH_2COOMe$ 15–19

S1. Synthesis of NHS- and MI-functionalized acyl chlorides

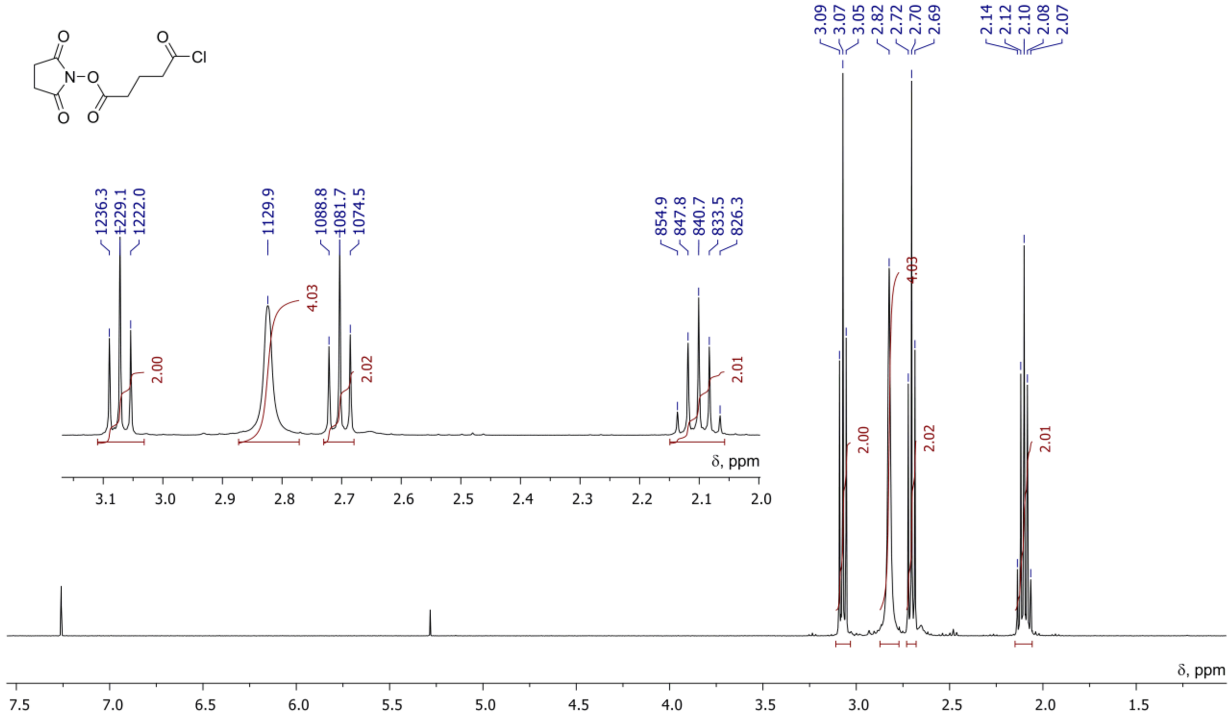

Figure S1. ^{1}H NMR spectrum (400 MHz, $CDCl_3$, 20 °C) of the acyl chloride **1**.

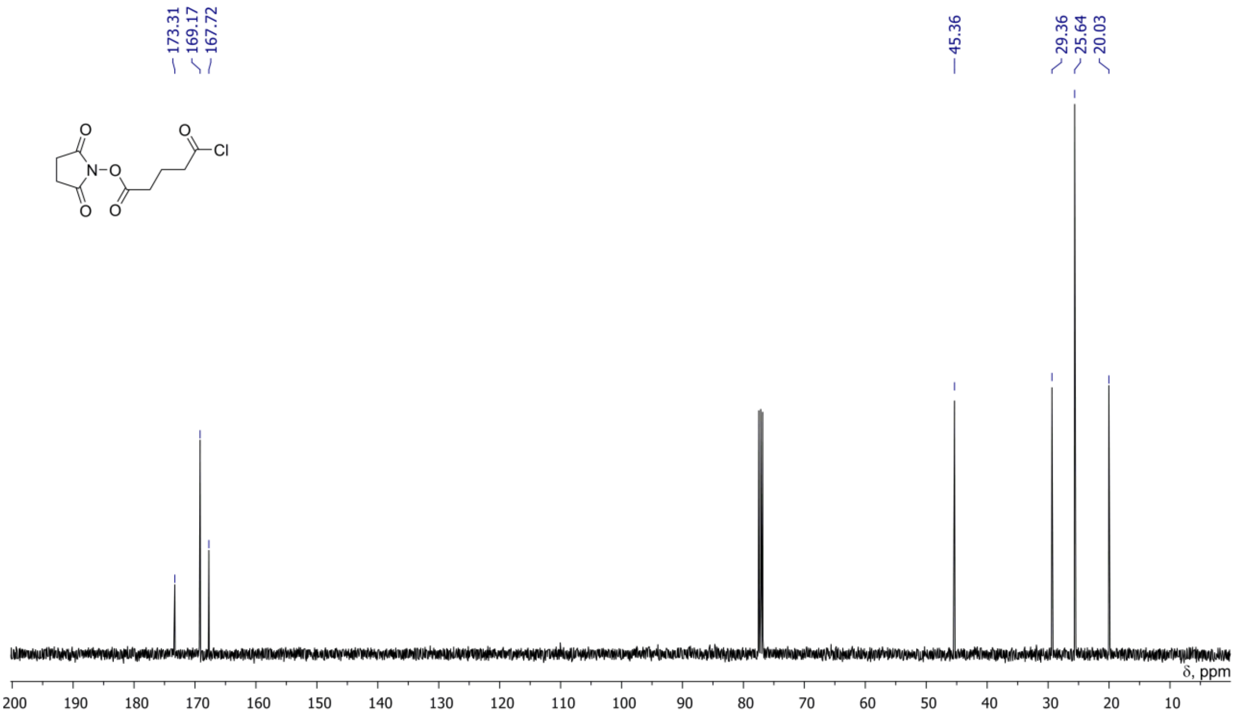

Figure S2. ^{13}C NMR spectrum (101 MHz, $CDCl_3$, 20 °C) of the acyl chloride **1**.

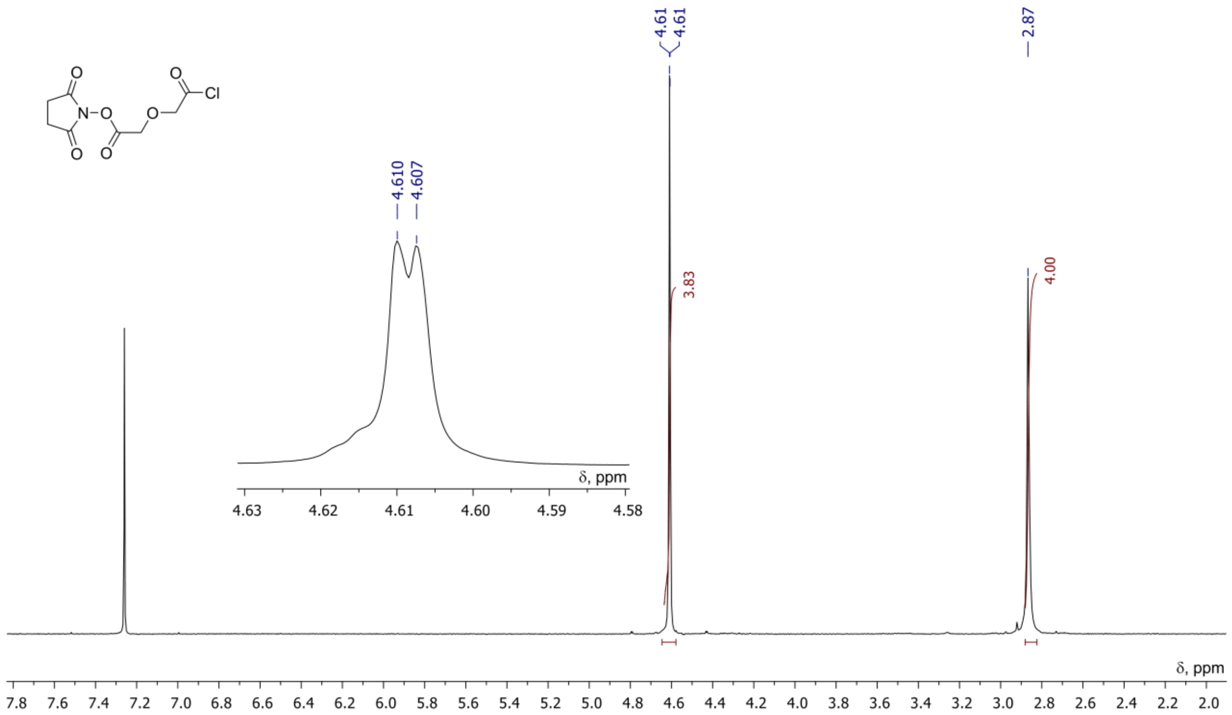

Figure S3. ^{1}H NMR spectrum (400 MHz, $CDCl_3$, 20 °C) of the acyl chloride **2**.

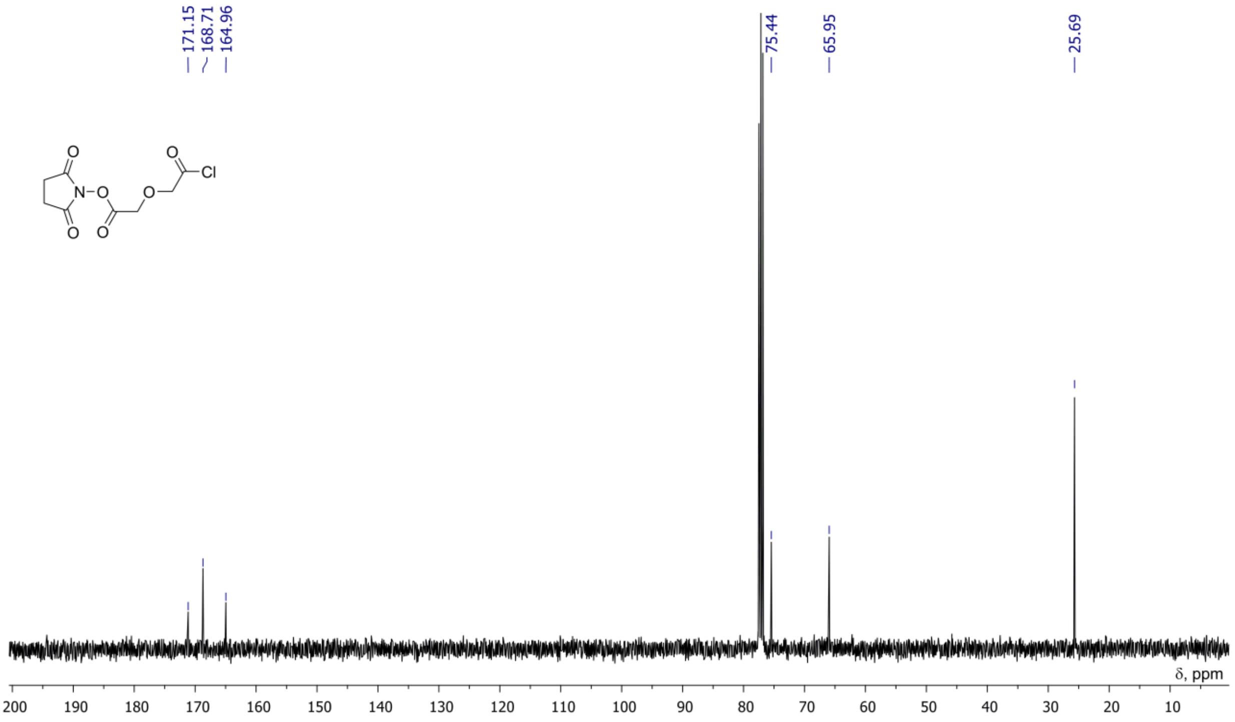

Figure S4. ^{13}C NMR spectrum (101 MHz, $CDCl_3$, 20 °C) of the acyl chloride **2**.

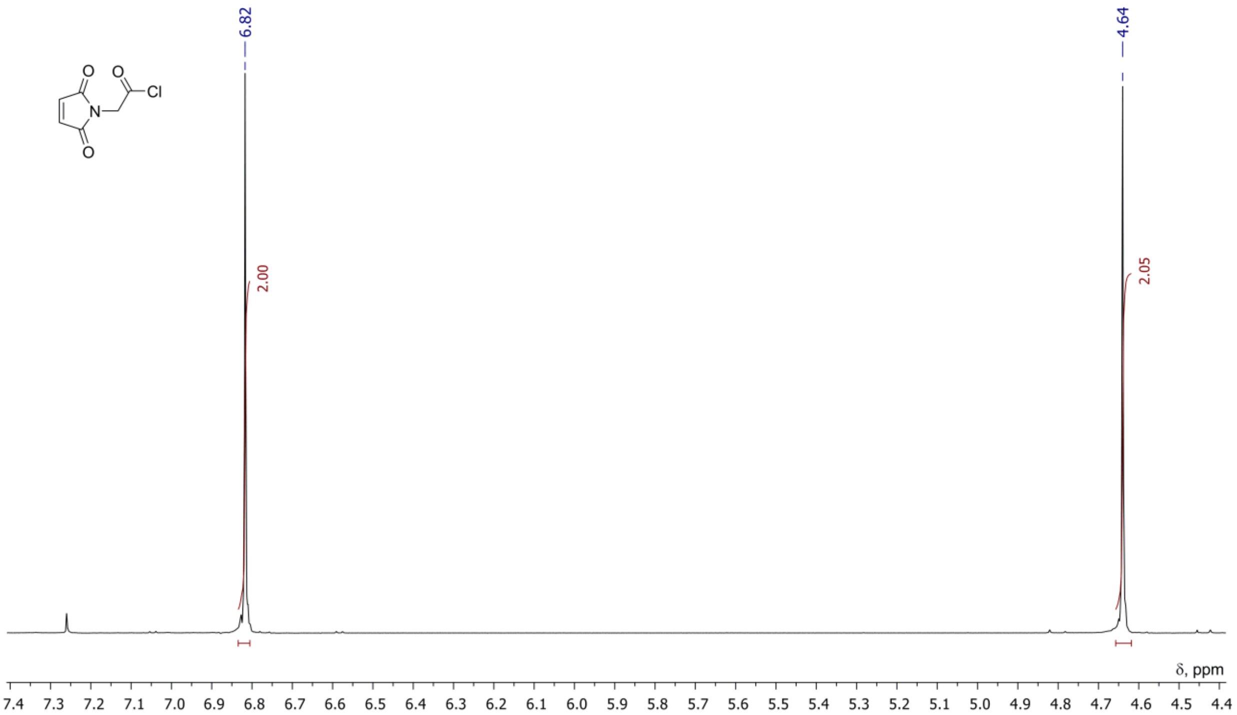

Figure S5. ^{1}H NMR spectrum (400 MHz, $CDCl_3$, 20 °C) of the acyl chloride **3**.

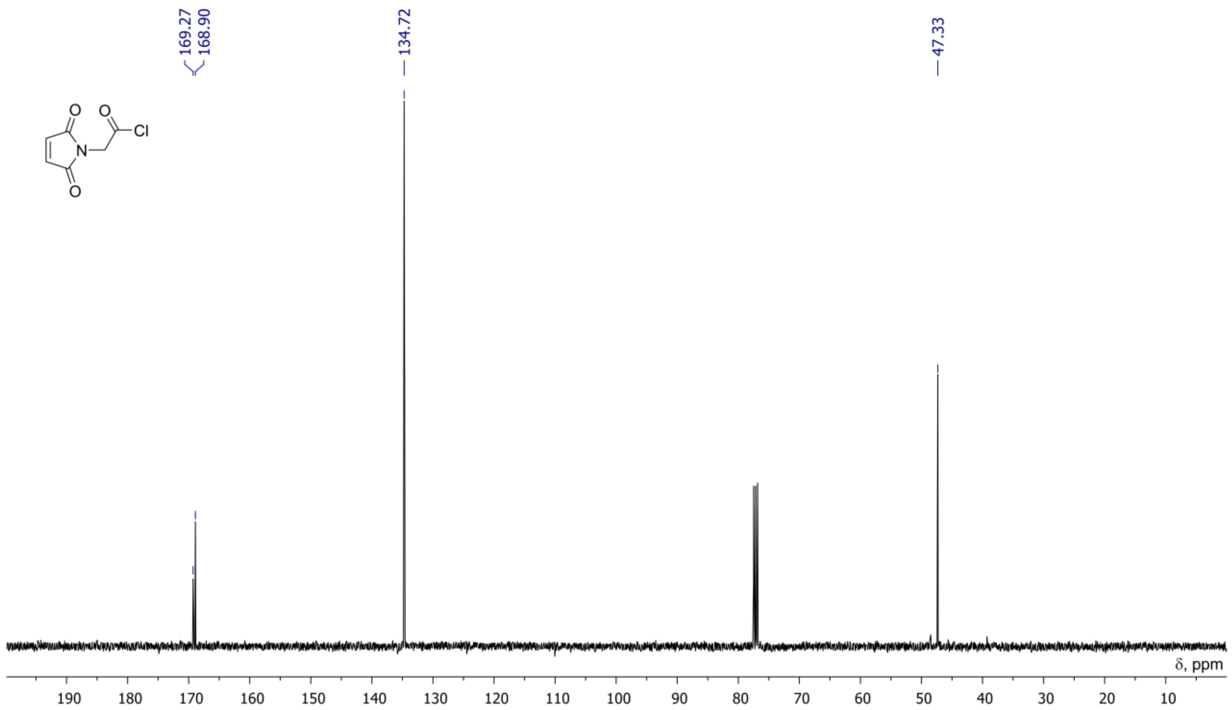

Figure S6. ^{13}C NMR spectrum (101 MHz, $CDCl_3$, 20 °C) of the acyl chloride **3**.

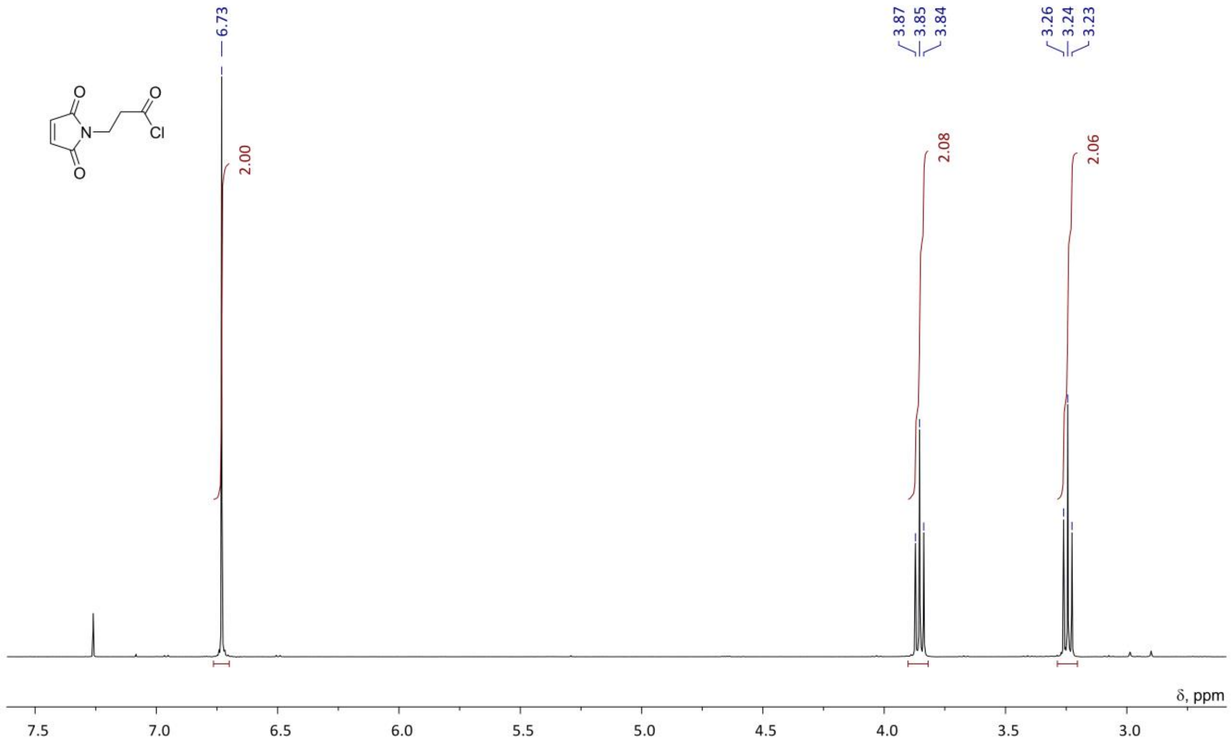

Figure S7. ^{1}H NMR spectrum (400 MHz, $CDCl_3$, 20 °C) of the acyl chloride **4**.

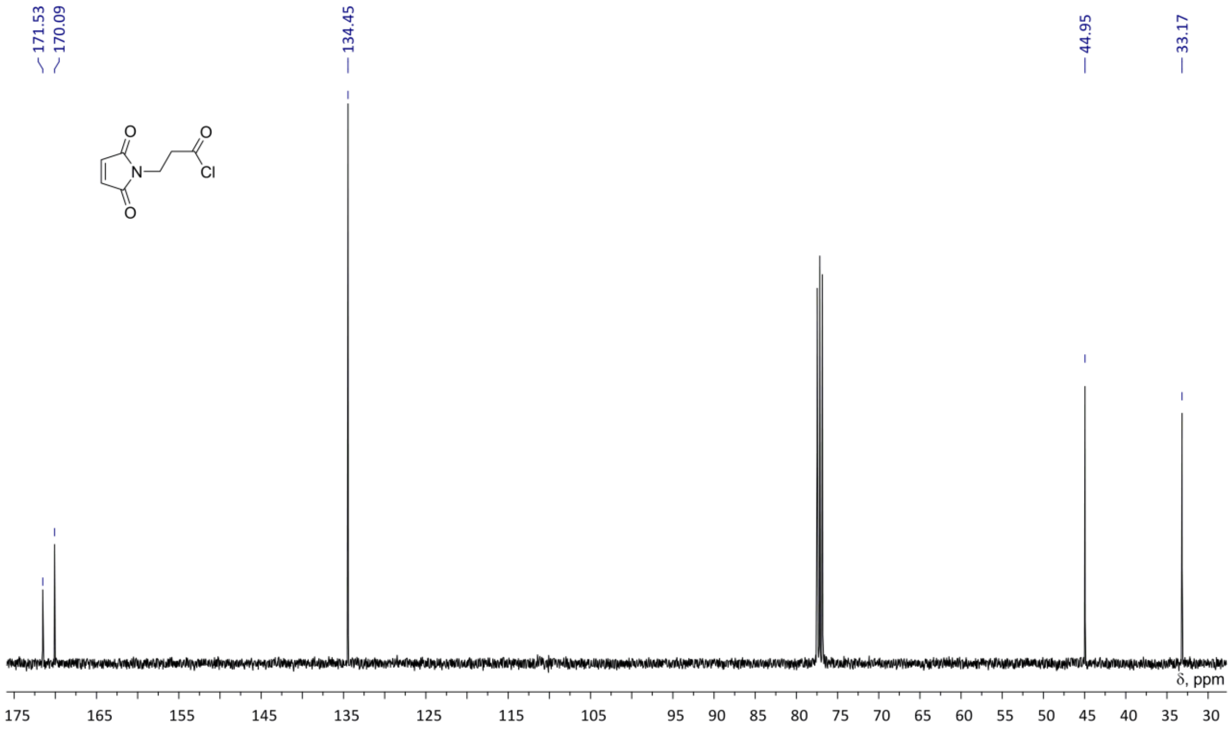

Figure S8. ^{13}C NMR spectrum (101 MHz, $CDCl_3$, 20 °C) of the acyl chloride **4**.

S2. Synthesis of functionalized polymers

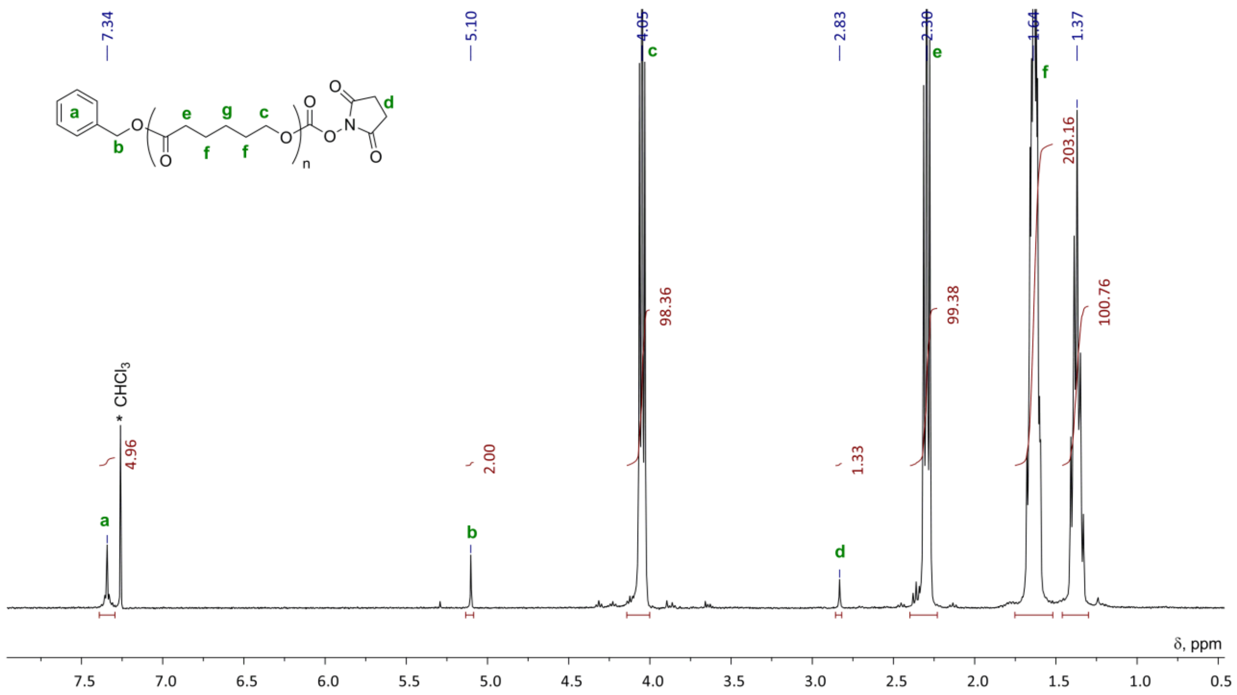

Figure S9. ^{1}H NMR spectrum (400 MHz, $CDCl_3$, 20 °C) of NHS-terminated poly(εCL) (Table 1, Entry 1).

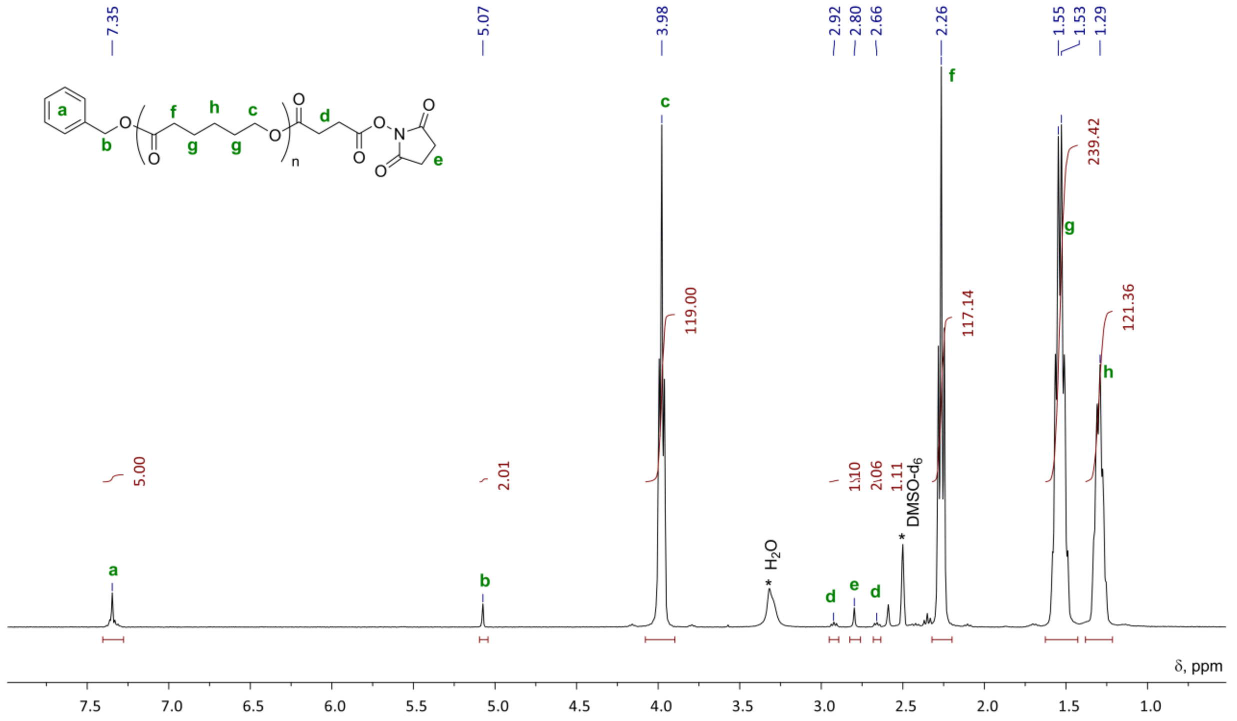

Figure S10. ^{1}H NMR spectrum (400 MHz, DMSO-d_6, 20 °C) of SA/NHS-terminated poly(εCL) (Table 1, Entry 2).

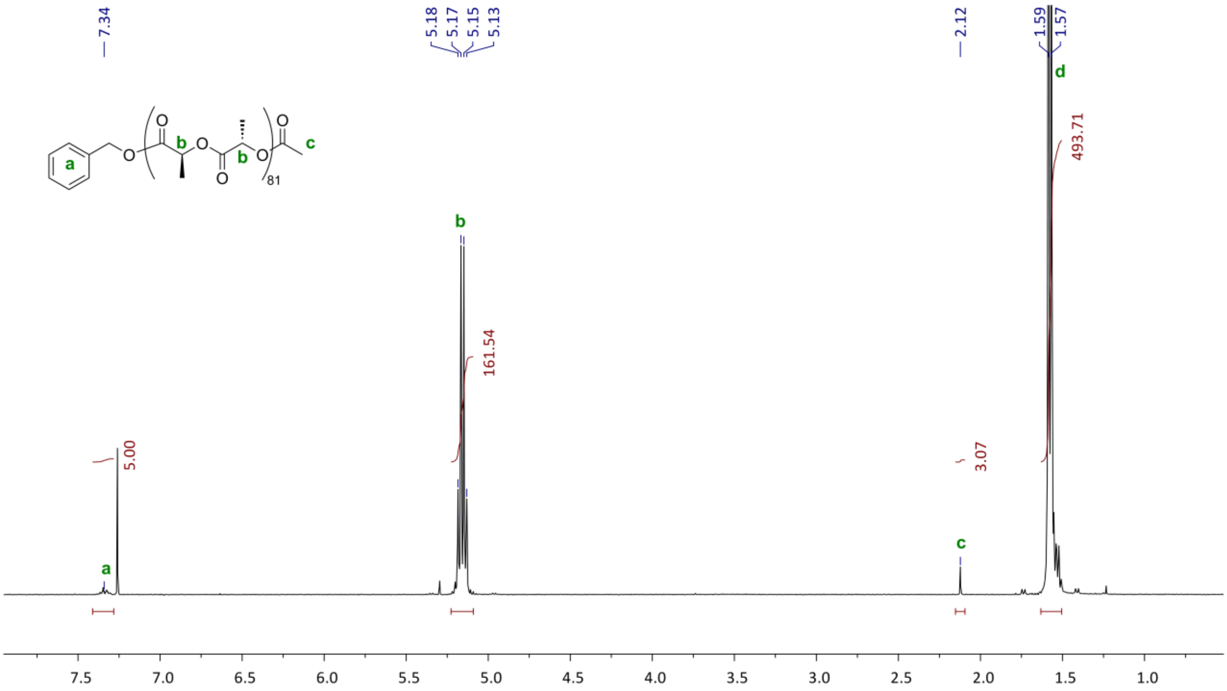

Figure S11. ^{1}H NMR spectrum (400 MHz, $CDCl_3$, 20 °C) of acetyl-terminated poly(*L*-LA).

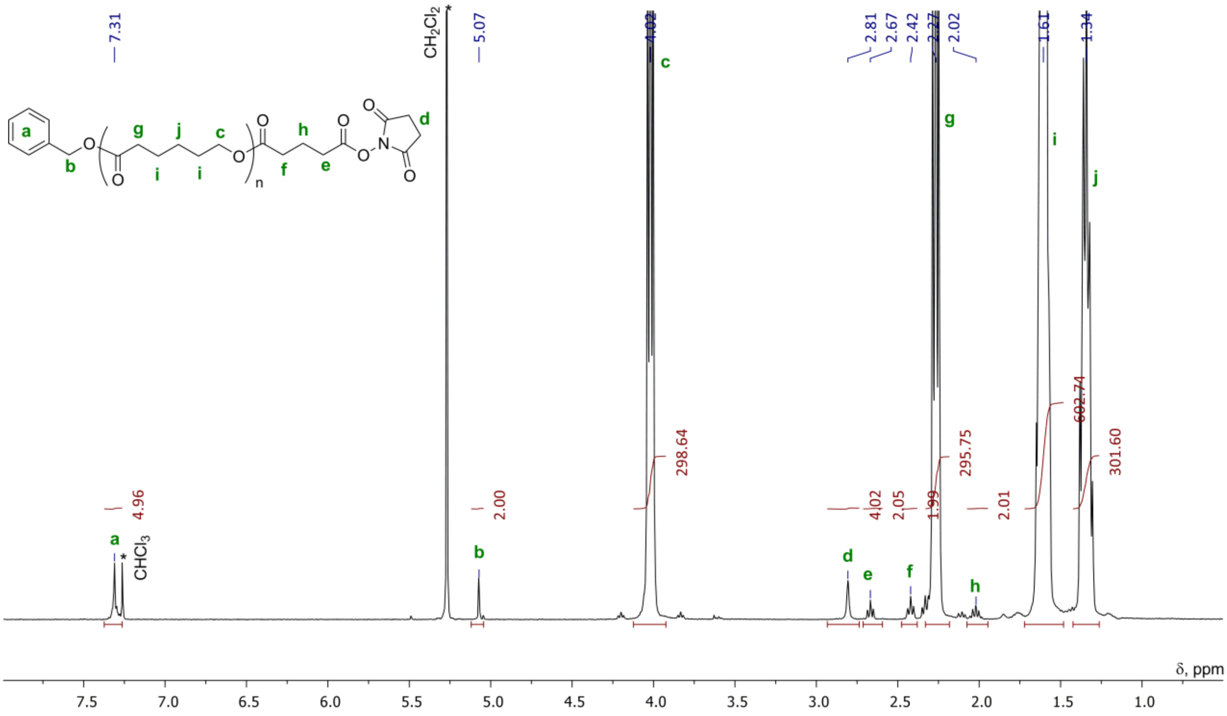

Figure S12. ^{1}H NMR spectrum (400 MHz, $CDCl_3$, 20 °C) of poly(εCL)-**1** (Table 1, Entry 3).

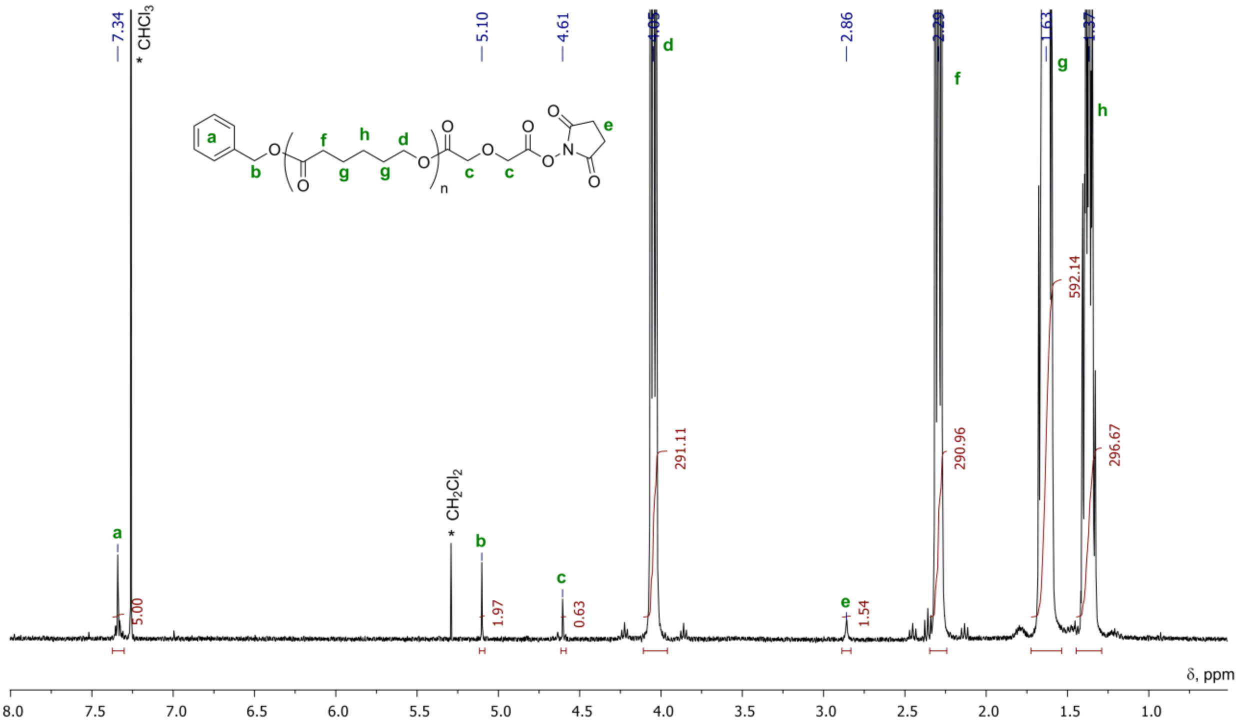

Figure S13. ^{1}H NMR spectrum (400 MHz, $CDCl_3$, 20 °C) of poly(εCL)-**2** (Table 1, Entry 4).

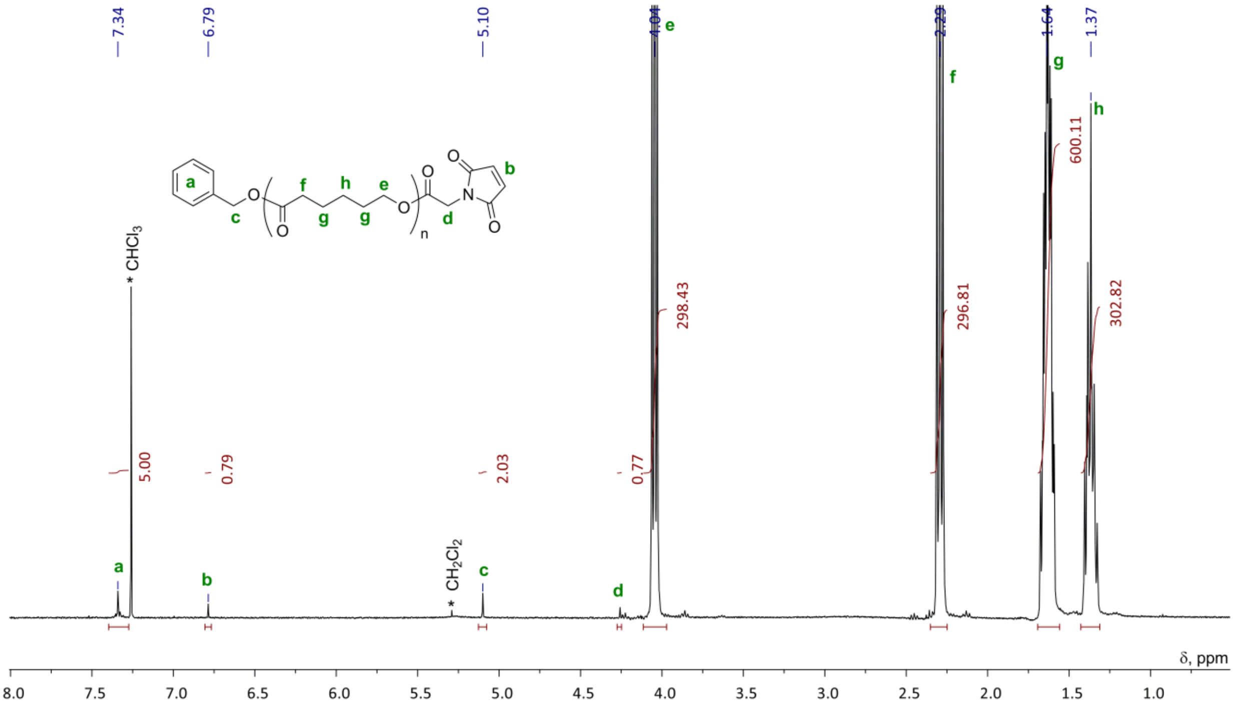

Figure S14. ^{1}H NMR spectrum (400 MHz, $CDCl_3$, 20 °C) of poly(εCL)-**3** (Table 1, Entry 5).

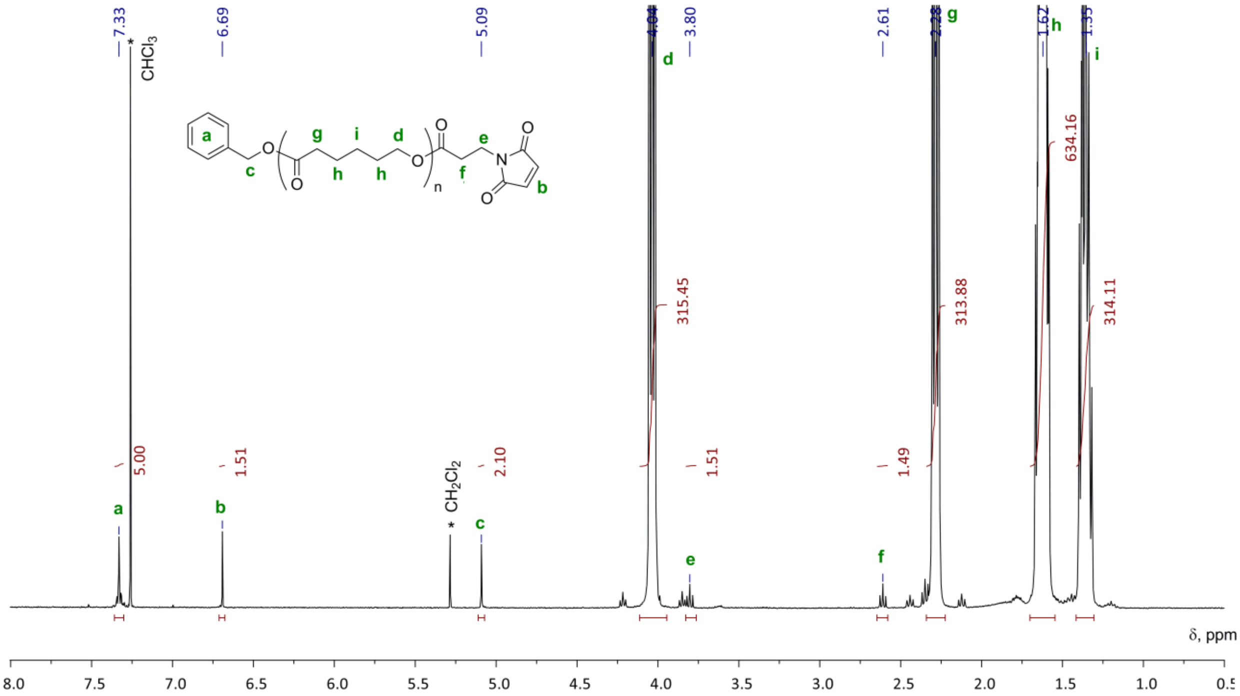

Figure S15. ^{1}H NMR spectrum (400 MHz, $CDCl_3$, 20 °C) of poly(εCL)-**4** (Table 1, Entry 6).

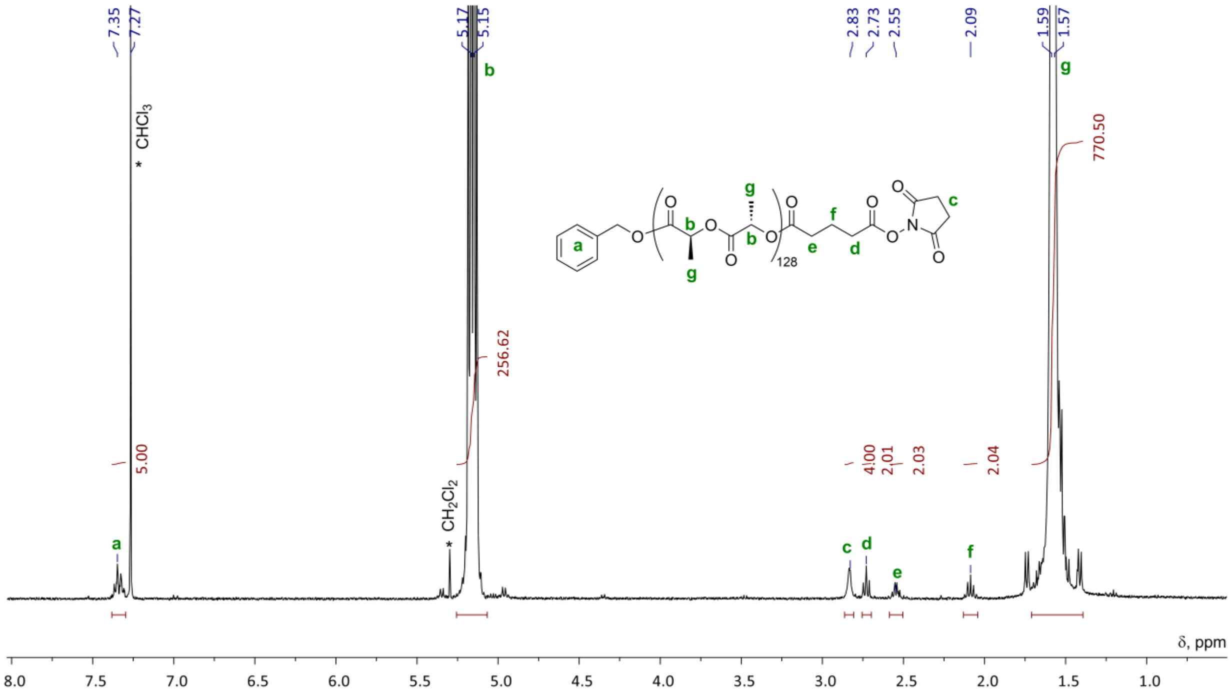

Figure S16. ^{1}H NMR spectrum (400 MHz, $CDCl_3$, 20 °C) of poly(*L*-LA)-**1** (Table 1, Entry 7).

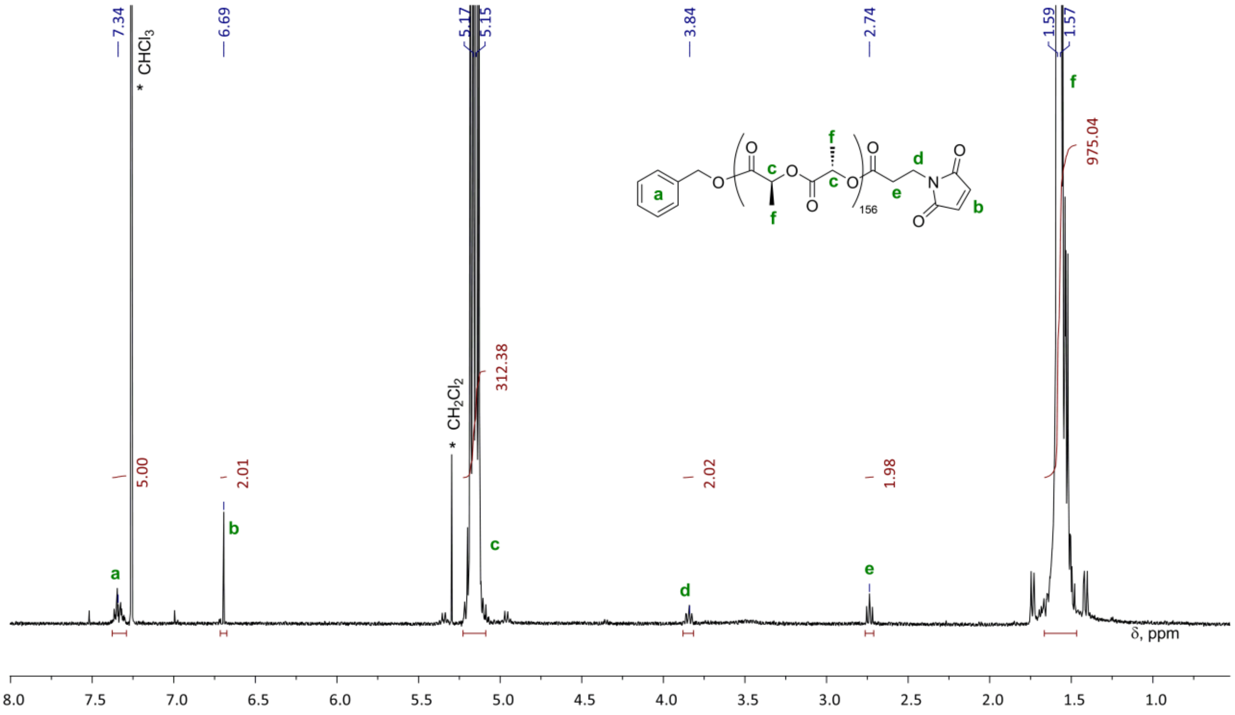

Figure S17. ^{1}H NMR spectrum (400 MHz, $CDCl_3$, 20 °C) of poly(*L*-LA)-**4** (Table 1, Entry 8).

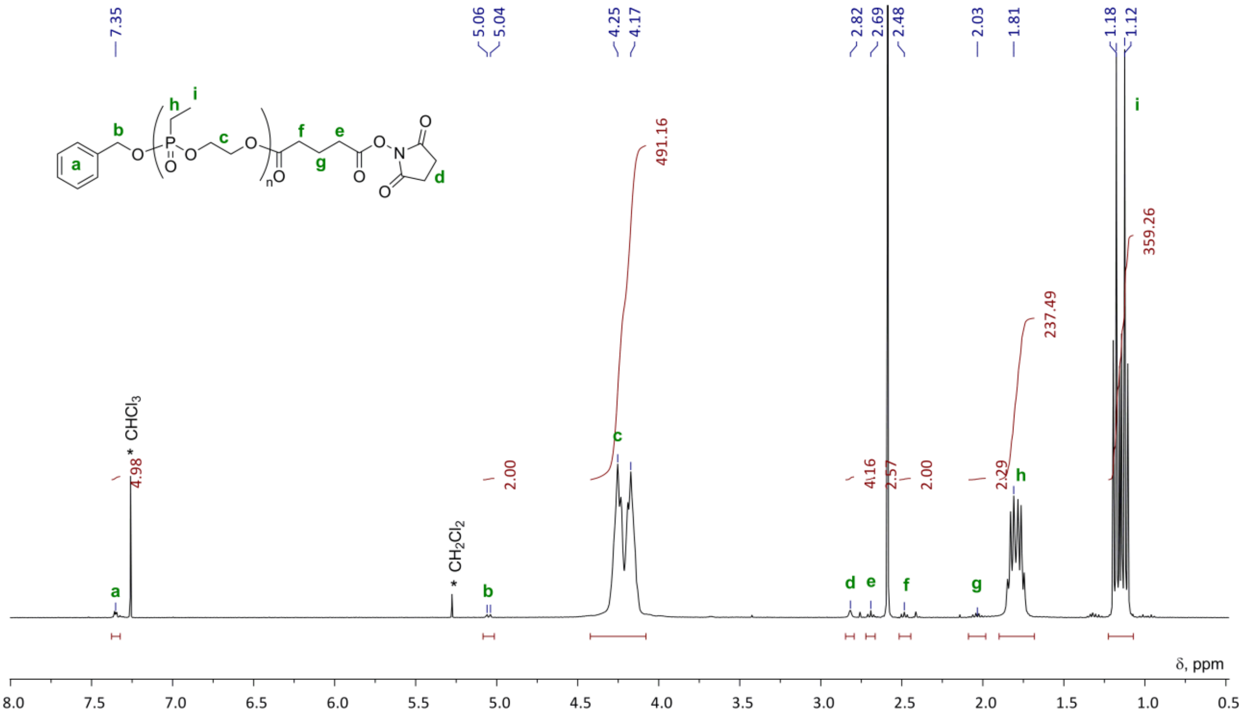

Figure S18. ^{1}H NMR spectrum (400 MHz, $CDCl_3$, 20 °C) of poly(EtEP)-**1** (Table 1, Entry 9).

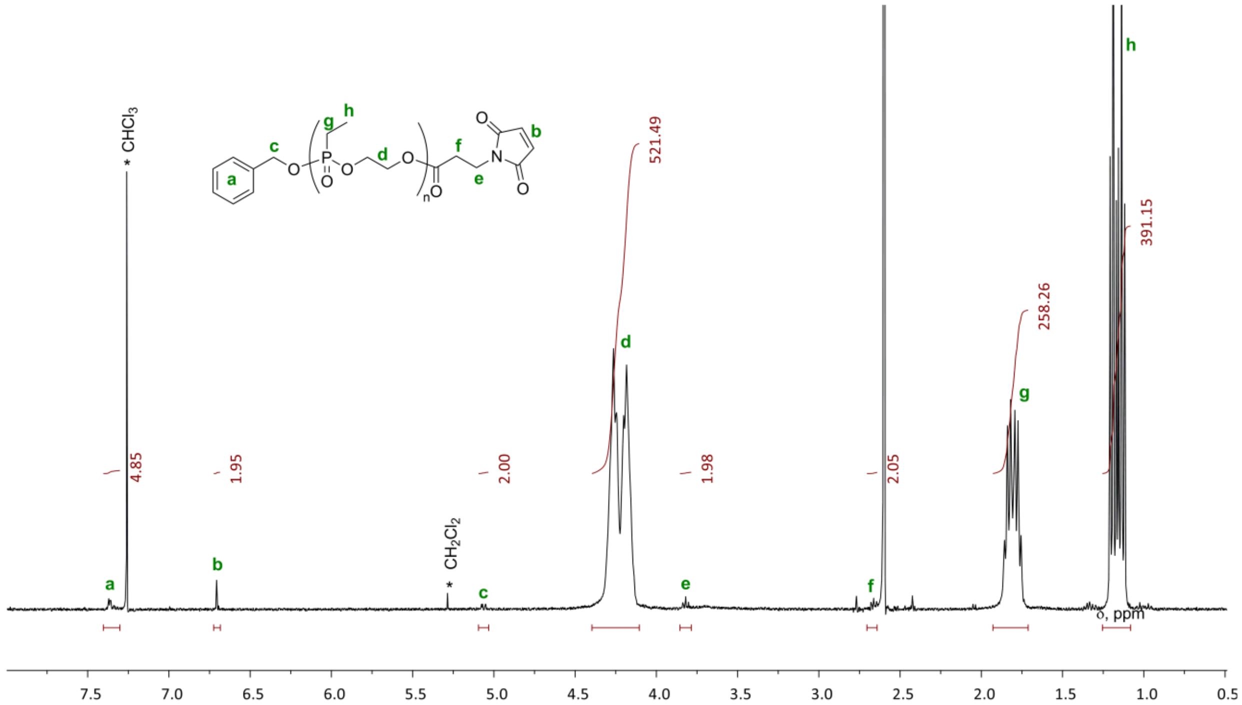

Figure S19. ^{1}H NMR spectrum (400 MHz, $CDCl_3$, 20 °C) of poly(EtEP)-**4** (Table 1, Entry 10).

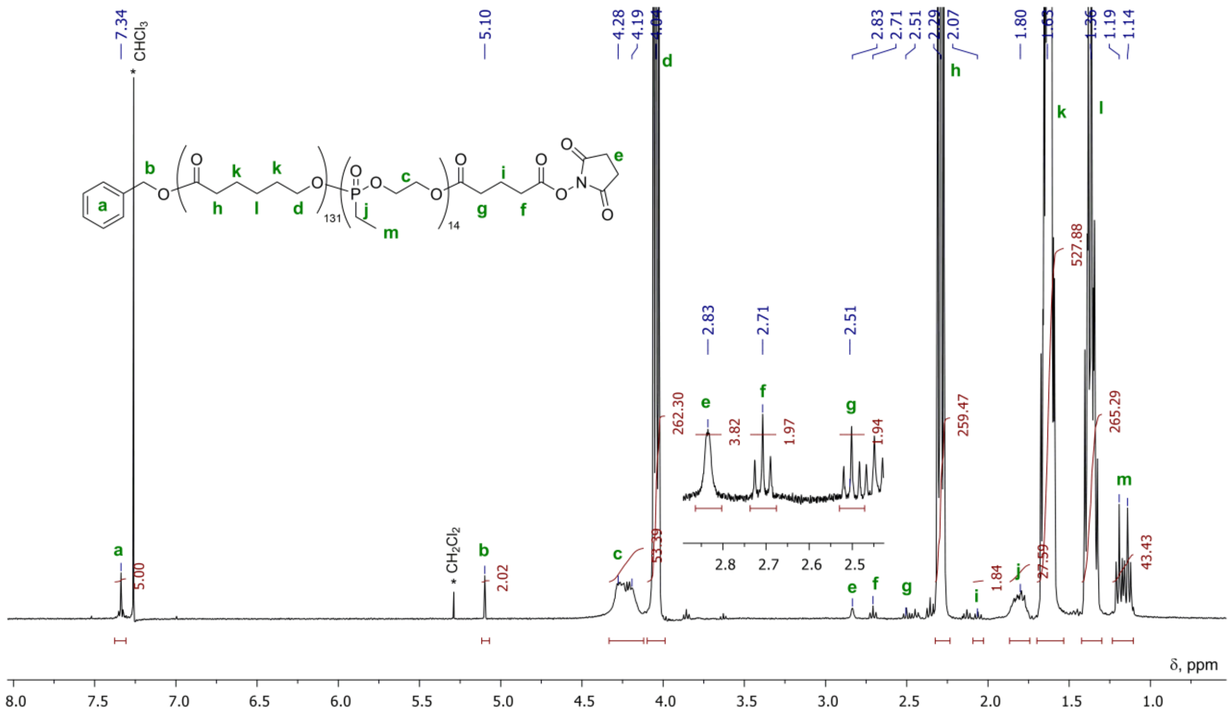

Figure S20. ^{1}H NMR spectrum (400 MHz, $CDCl_3$, 20 °C) of poly(εCL)-*b*-poly(EtEP)-**1** (Table 1, Entry 11).

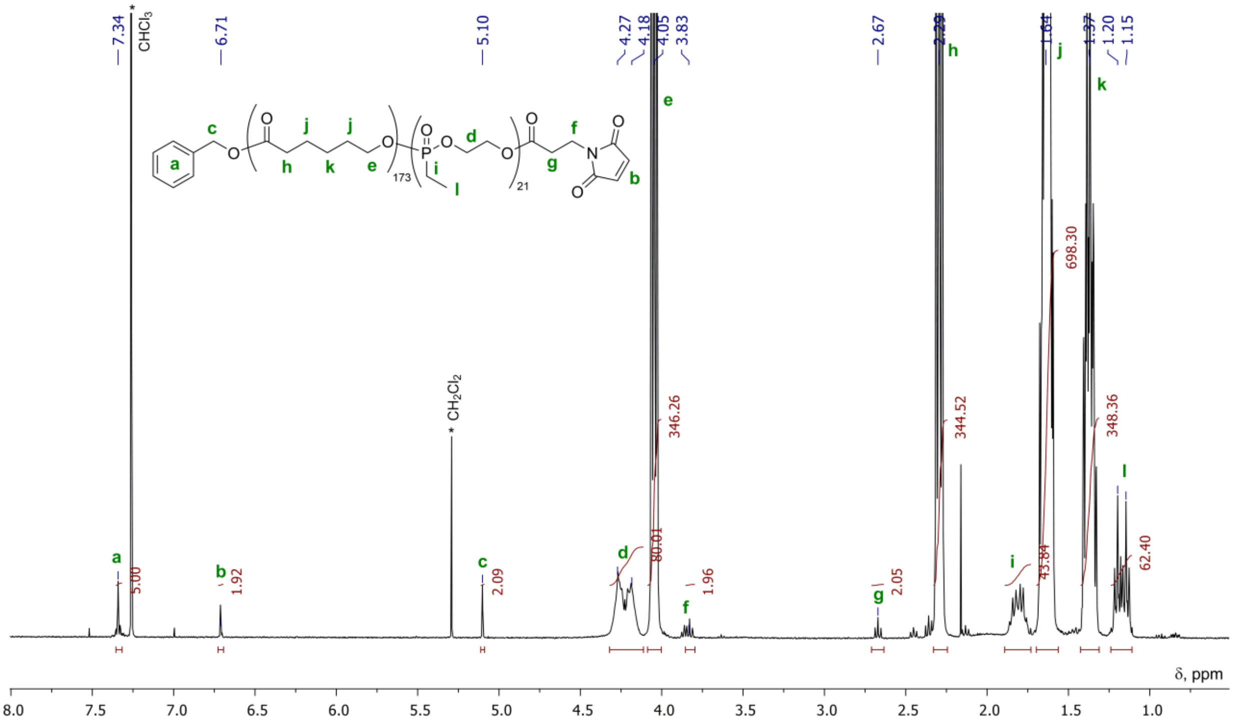

Figure S21. ^{1}H NMR spectrum (400 MHz, $CDCl_3$, 20 °C) of poly(εCL)-*b*-poly(EtEP)-**4** (Table 1, Entry 12).

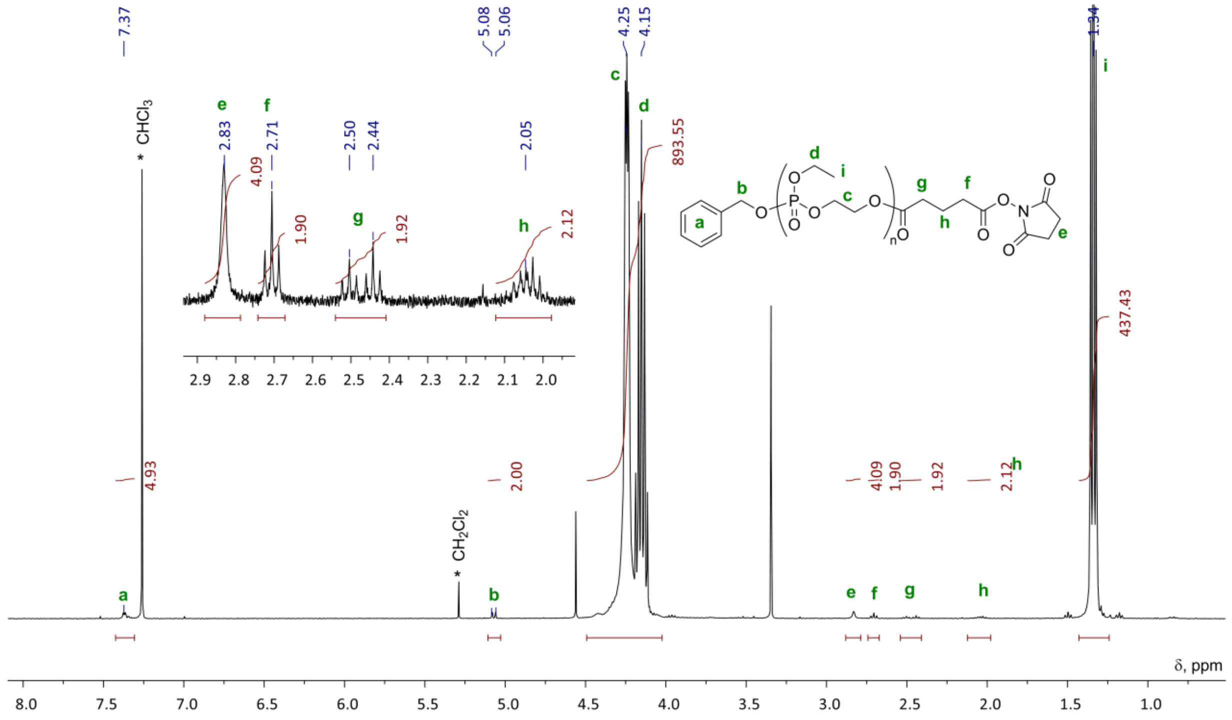

Figure S22. ^{1}H NMR spectrum (400 MHz, $CDCl_3$, 20 °C) of poly(EtOEP)-**1** (Table 1, Entry 13).

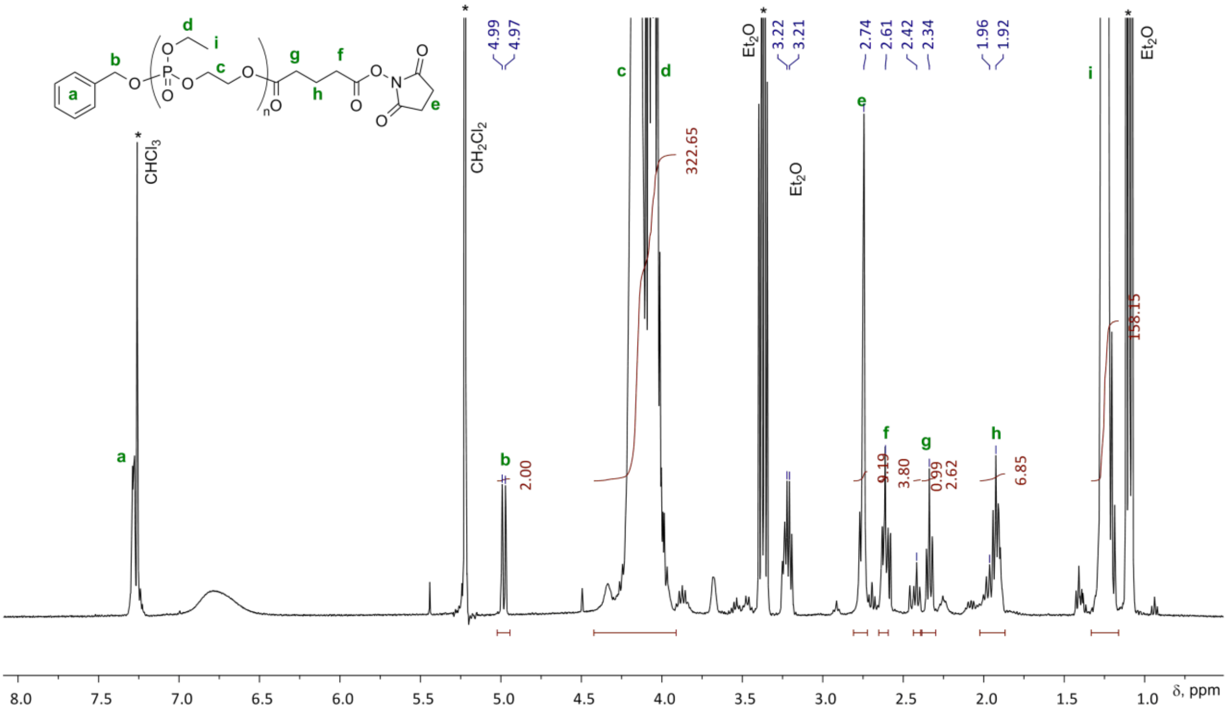

Figure S23. ^{1}H NMR spectrum (400 MHz, $CDCl_3$, 20 °C) of poly(EtOEP)-**1** obtained usingTBD/BnOH initiation.

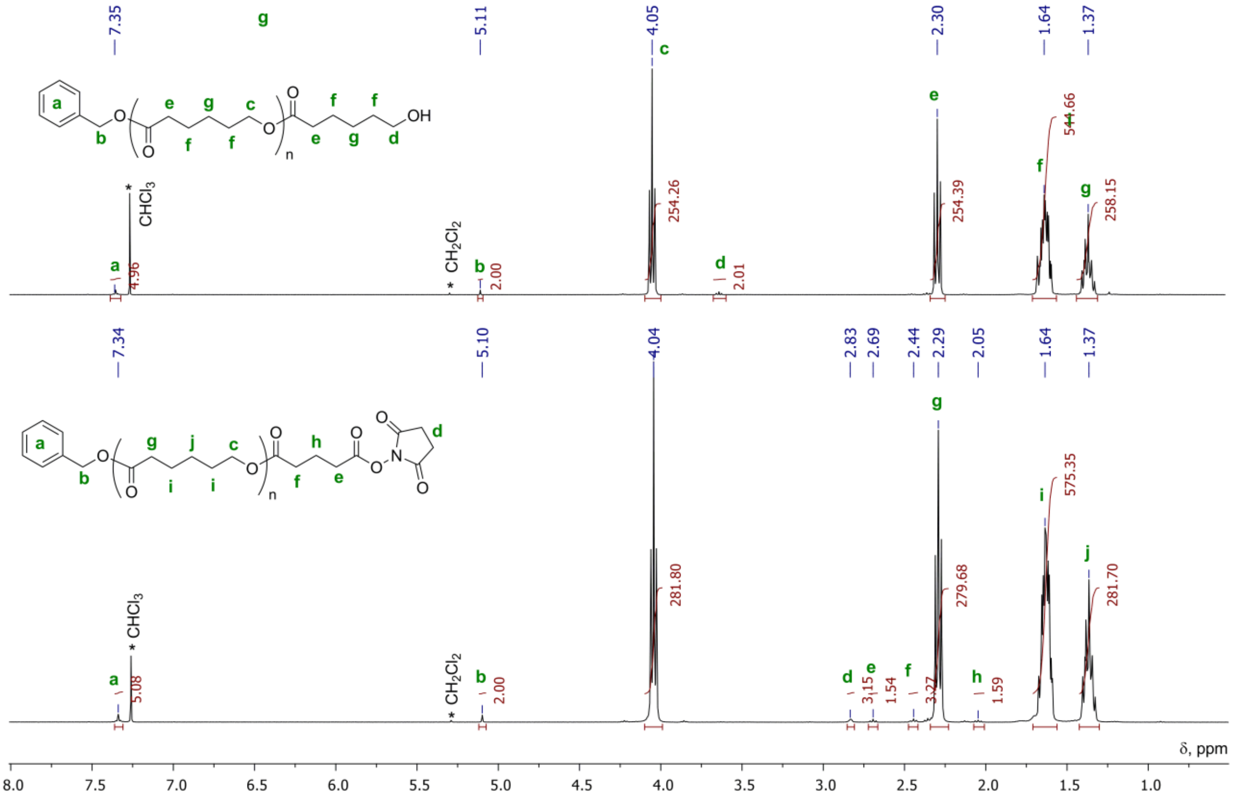

Figure S24. ^{1}H NMR spectrum (400 MHz, $CDCl_3$, 20 °C) of NHS-functionalized poly(εCL) obtained by the reaction of acyl chloride **1** with poly(εCL).

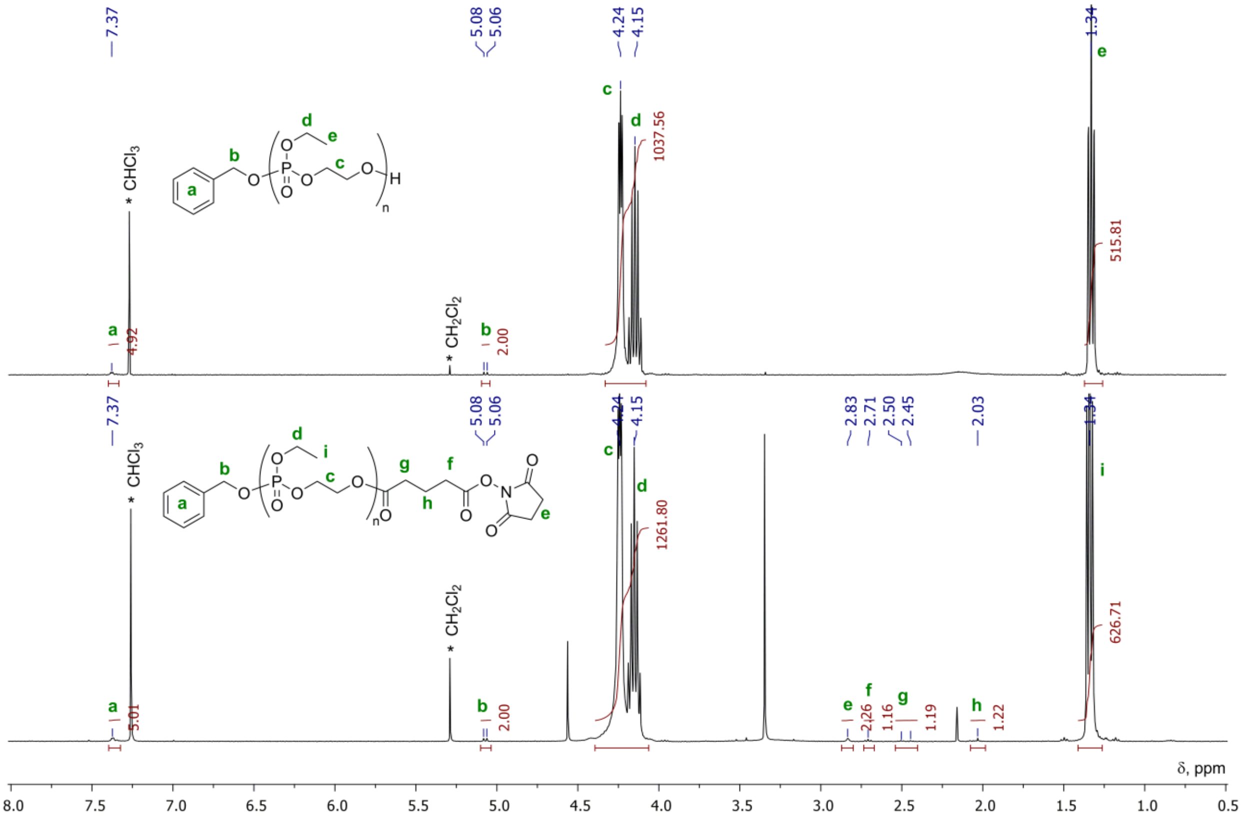

Figure S25. ^{1}H NMR spectrum (400 MHz, $CDCl_3$, 20 °C) of NHS-functionalized poly(EtOEP) obtained by the reaction of acyl chloride **1** with poly(EtOEP).

S3. Reactions of functionalized polymers with $^{i}BuNH_2$ and $HSCH_2COOMe$

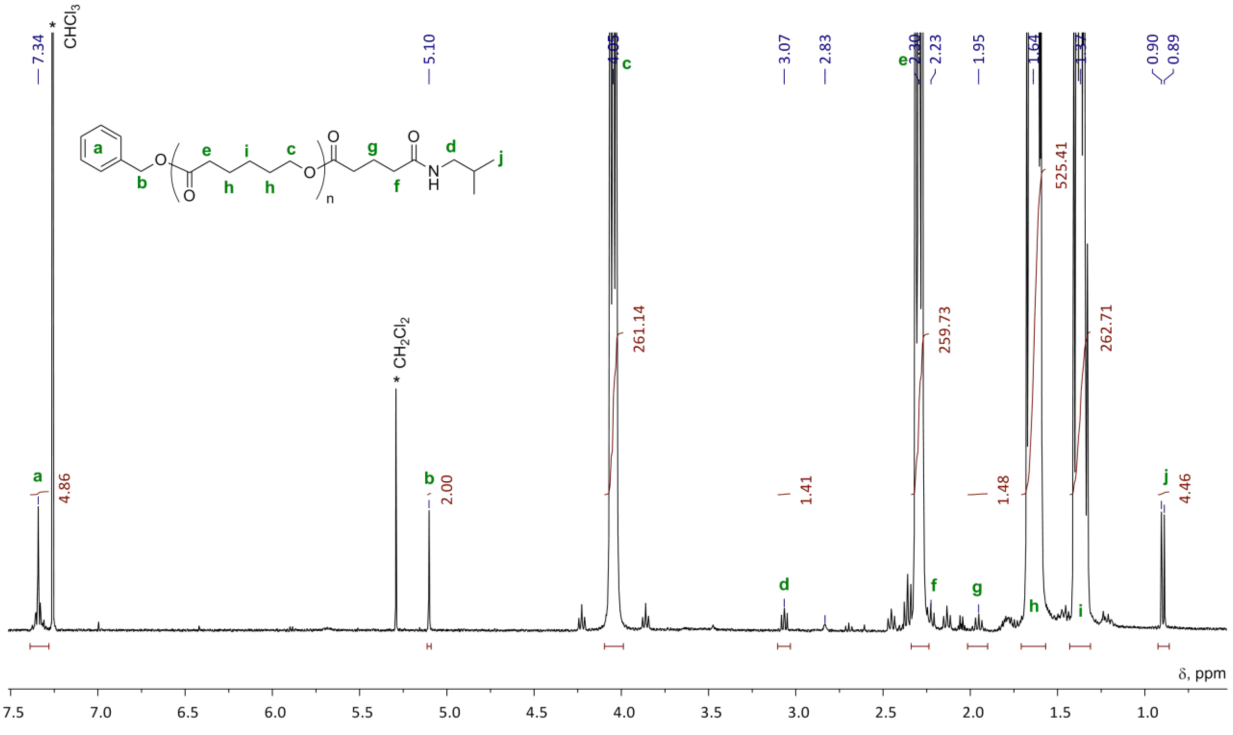

Figure S26. ^{1}H NMR spectrum (400 MHz, $CDCl_3$, 20 °C) of poly(εCL)-**1**-N.

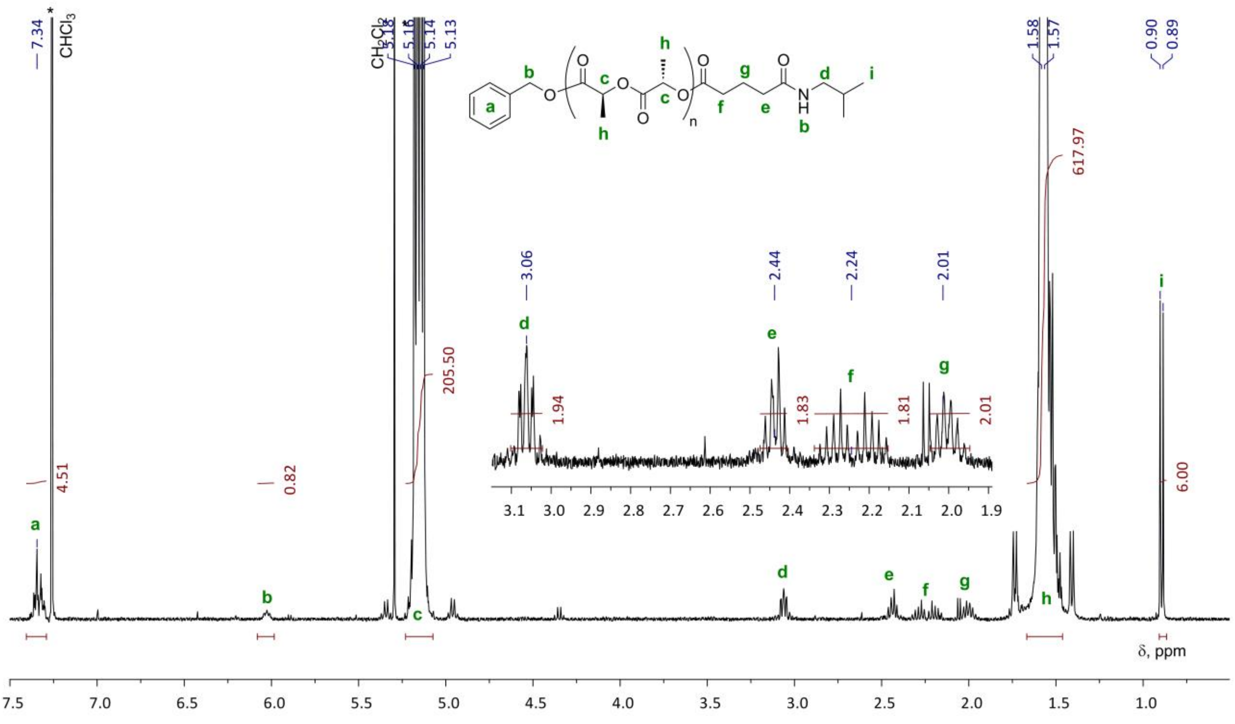

Figure S27. ^{1}H NMR spectrum (400 MHz, $CDCl_3$, 20 °C) of poly(*L*-LA)-**1**-N.

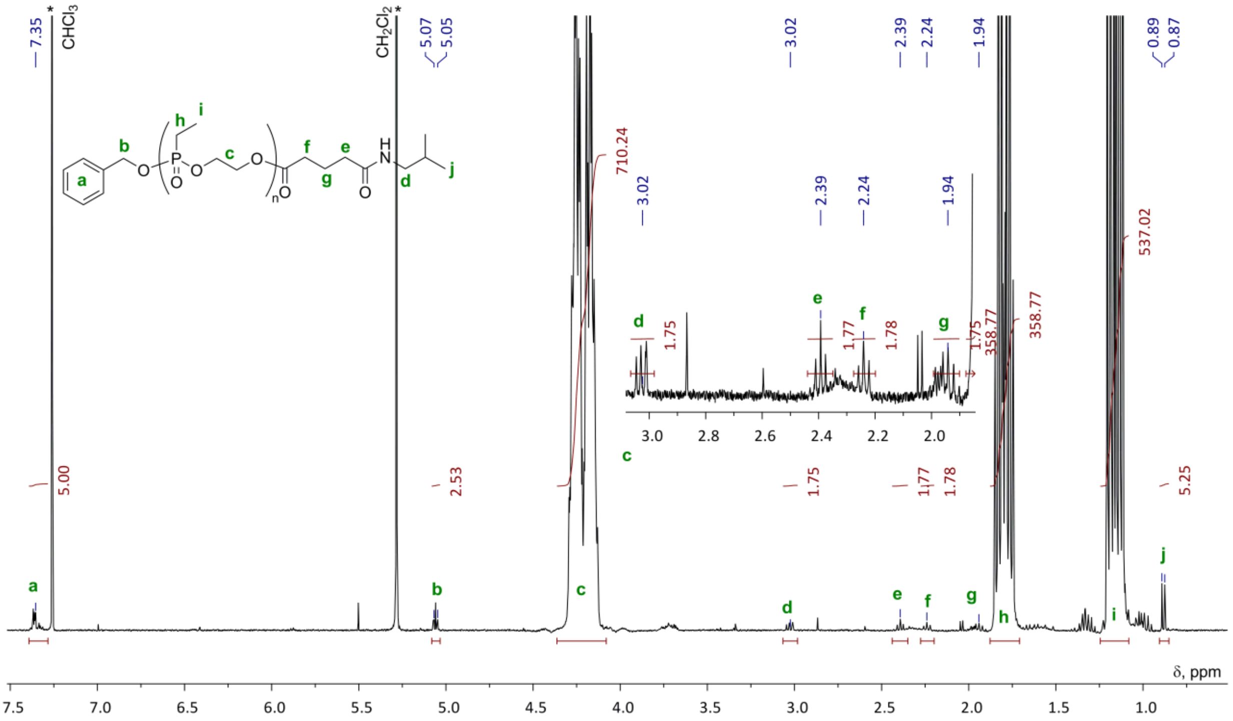

Figure S28. ^{1}H NMR spectrum (400 MHz, $CDCl_3$, 20 °C) of poly(EtEP)-**1**-N.

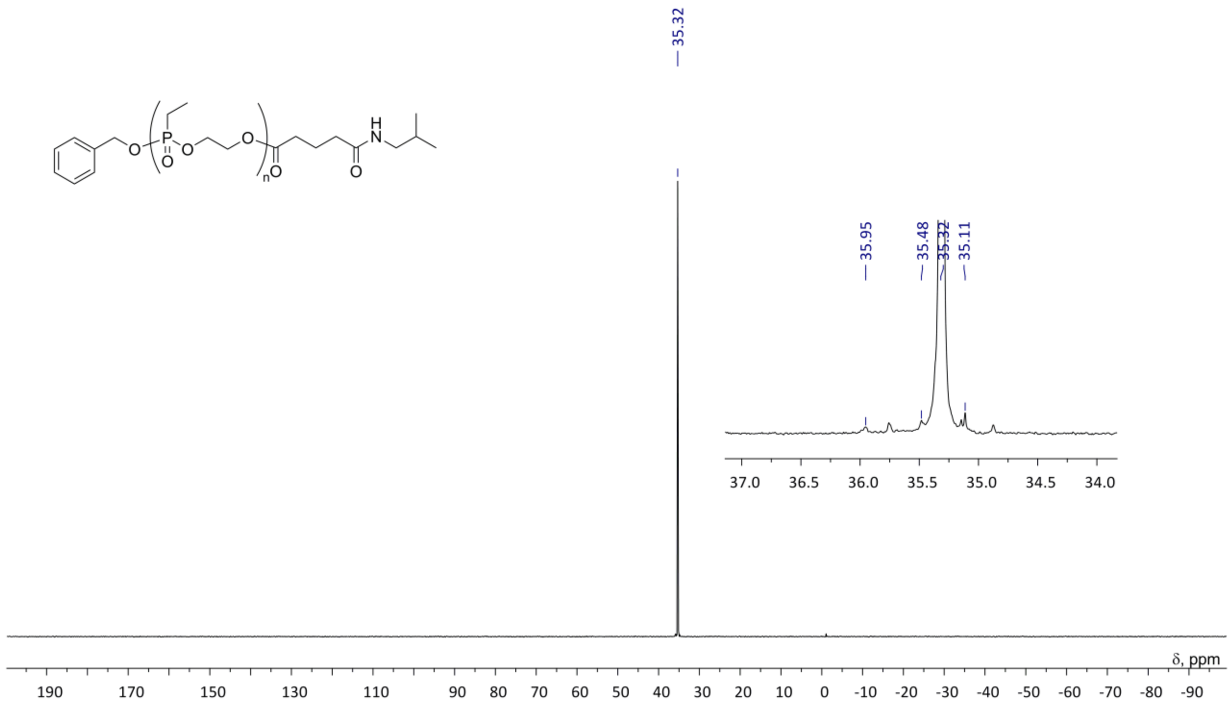

Figure S29. ^{31}P NMR spectrum (162 MHz, $CDCl_3$, 20 °C) of poly(EtEP)-**1**-N.

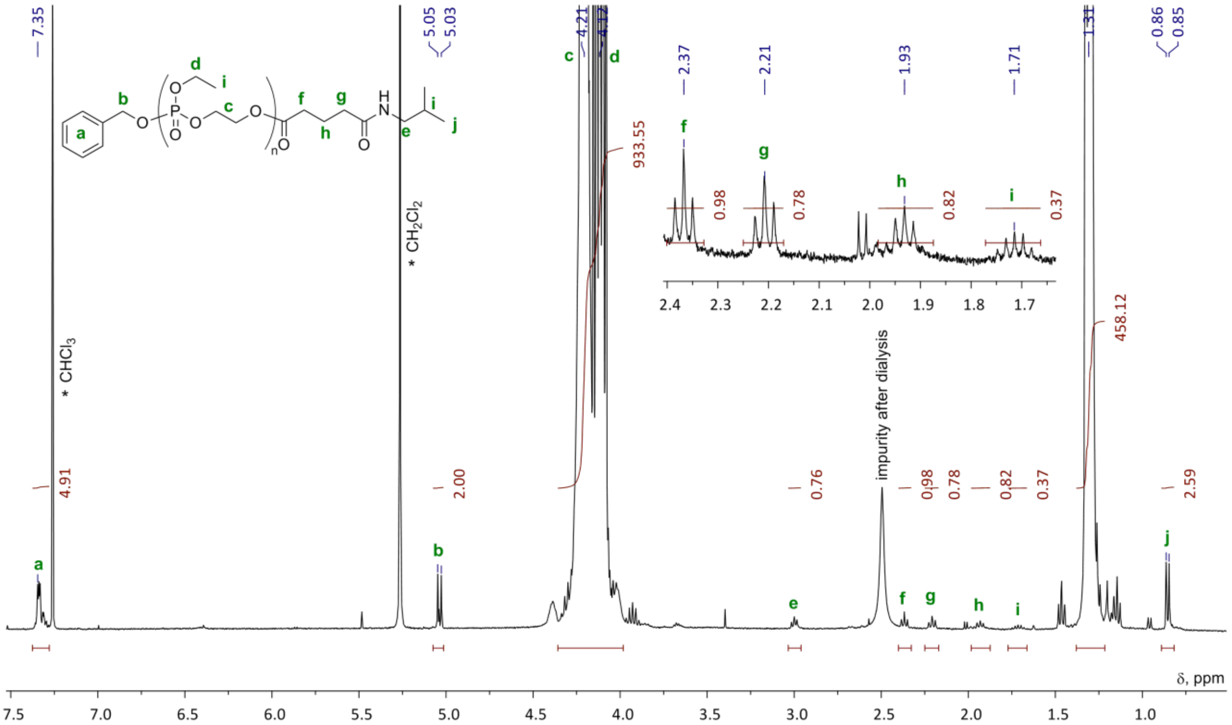

Figure S30. ^{1}H NMR spectrum (400 MHz, $CDCl_3$, 20 °C) of poly(EtOEP)-**1**-N.

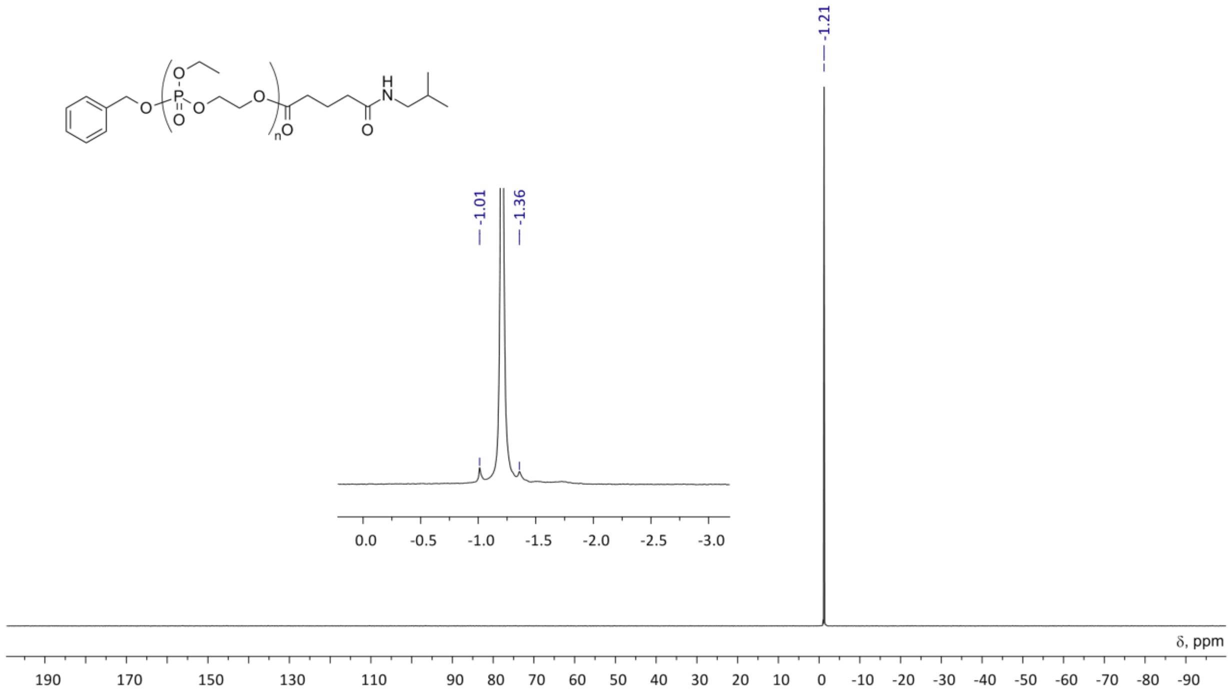

Figure S31. ^{31}P NMR spectrum (162 MHz, $CDCl_3$, 20 °C) of poly(EtOEP)-**1**-N.

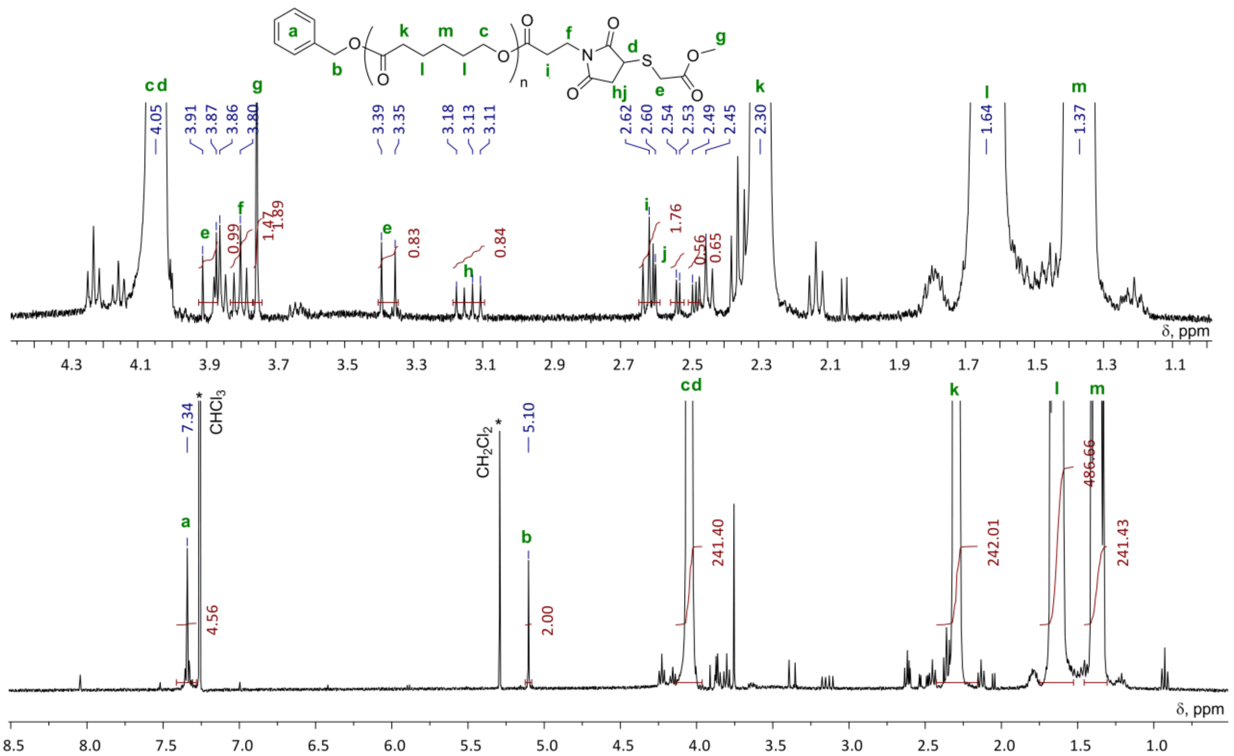

Figure S32. ^{1}H NMR spectrum (400 MHz, $CDCl_3$, 20 °C) of poly(εCL)-**4**-S.

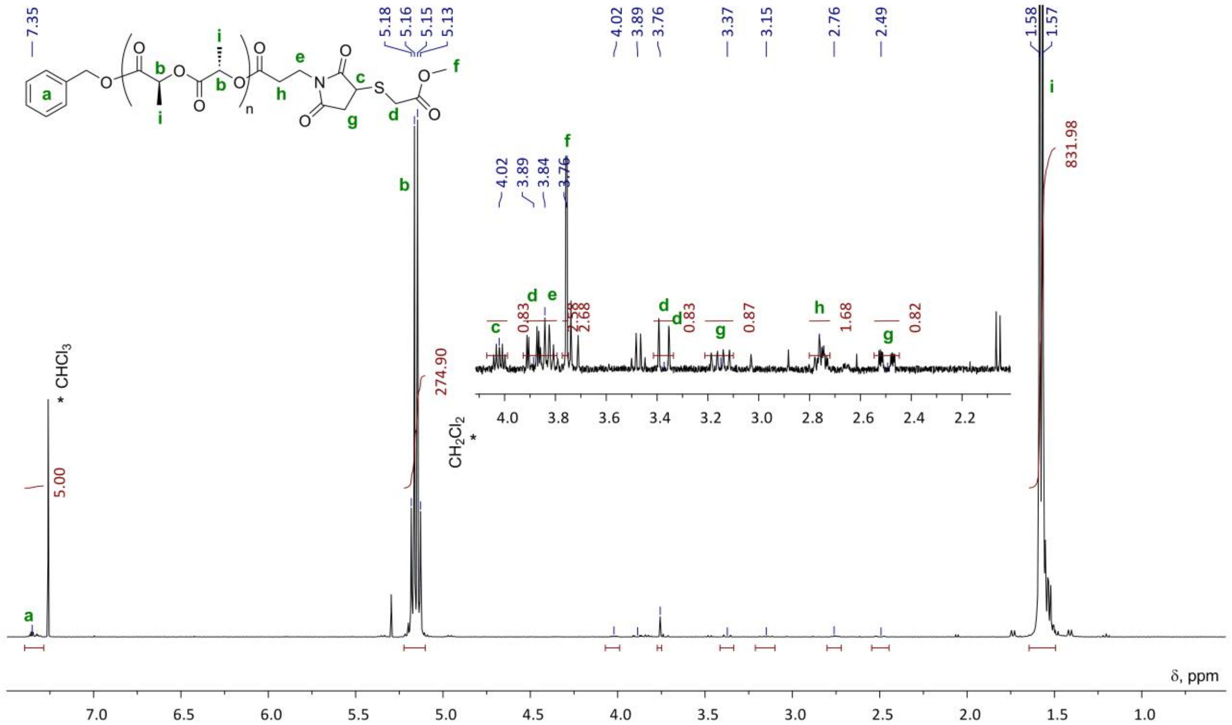

Figure S33. ^{1}H NMR spectrum (400 MHz, $CDCl_3$, 20 °C) of poly(*L*-LA)-**4**-S.

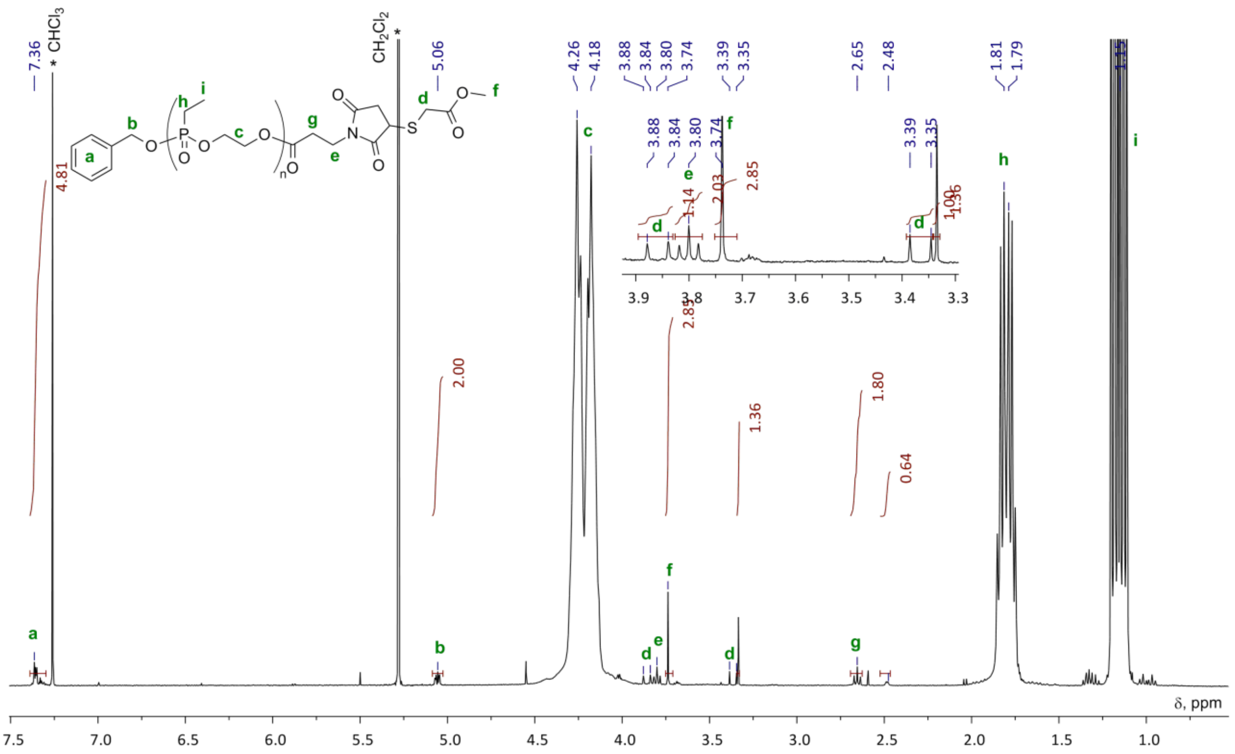

Figure S34. ^{1}H NMR spectrum (400 MHz, $CDCl_3$, 20 °C) of poly(EtEP)-**4**-S.

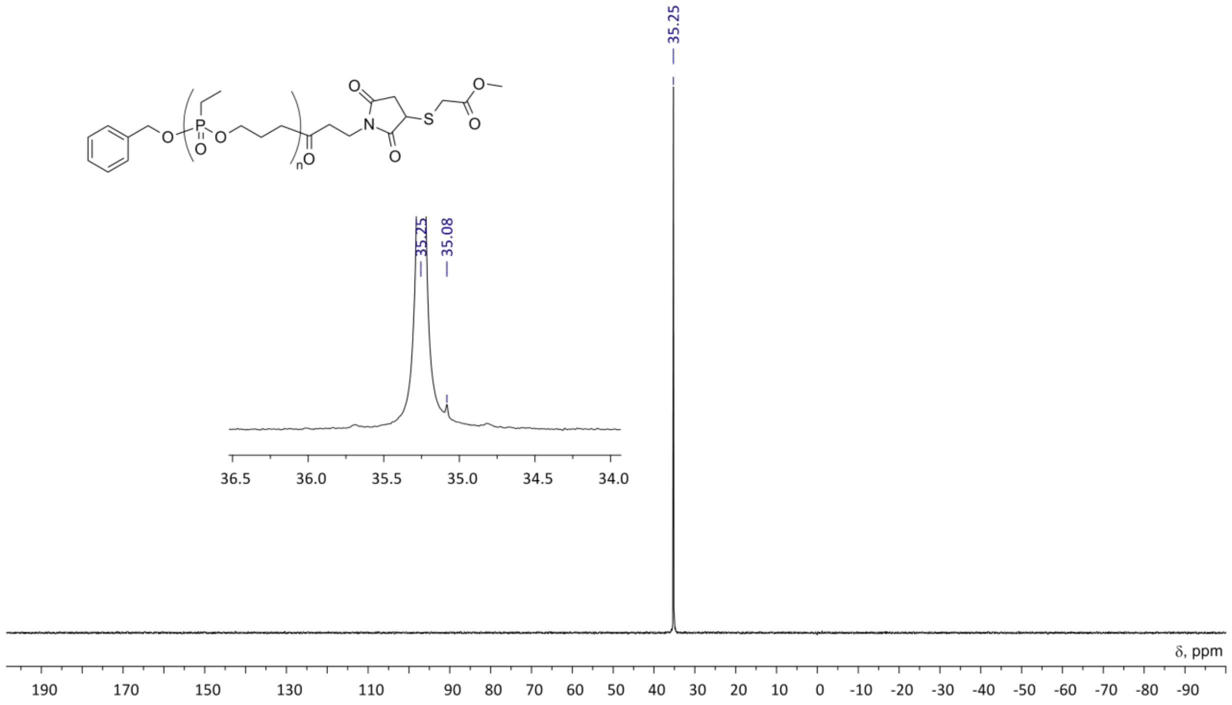

Figure S35. ^{31}P NMR spectrum (162 MHz, $CDCl_3$, 20 °C) of poly(EtEP)-**4**-S.
